# Increased serum QUIN/KYNA is a reliable biomarker of post-stroke cognitive decline

**DOI:** 10.1186/s13024-020-00421-4

**Published:** 2021-02-15

**Authors:** Adrien Cogo, Gabrielle Mangin, Benjamin Maïer, Jacques Callebert, Mikael Mazighi, Hughes Chabriat, Jean-Marie Launay, Gilles Huberfeld, Nathalie Kubis

**Affiliations:** 1 Université de Paris, INSERM U1148, Laboratory for Vascular Translational Science, F-75018 Paris, France; 2 Université de Paris, INSERM U965, CART, F-75010 Paris, France; 3 Université de Paris, Inserm UMR-S 942; Département de Biochimie et de Biologie Moléculaire, APHP, Hôpital Lariboisière, F-75010 Paris, France; 4 Service de Neurologie, APHP, Hôpital Lariboisière, F-75010 Paris, France; 5 Neuroglial Interactions in Cerebral Physiopathology, Center for Interdisciplinary Research in Biology, Collège de France, CNRS UMR 7241, INSERM U1050, Labex Memolife, PSL Research University, F-75005 Paris, France; 6 Clinical Neurophysiology department, APHP, Pitie-Salpetriere Hospital, Sorbonne Université, APHP, F-75013 Paris, France; 7 Service de Physiologie Clinique-Explorations Fonctionnelles, DMU DREAM, APHP, Hôpital Lariboisière, F-75010 Paris, France

**Keywords:** Post-stroke dementia • mouse • electrophysiology • tryptophane • cerebral ischemia • indolamine 2, 3-dioxygenase

## Abstract

**Background:**

Strokes are becoming less severe due to increased numbers of intensive care units and improved treatments. As patients survive longer, post-stroke cognitive impairment (PSCI) has become a major health public issue. Diabetes has been identified as an independent predictive factor for PSCI. Here, we characterized a clinically relevant mouse model of PSCI, induced by permanent cerebral artery occlusion in diabetic mice, and investigated whether a reliable biomarker of PSCI may emerge from the kynurenine pathway which has been linked to inflammatory processes.

**Methods:**

Cortical infarct was induced by permanent middle cerebral artery occlusion in male diabetic mice (streptozotocin IP). Six weeks later, cognitive assessment was performed using the Barnes maze, hippocampi long-term potentiation using microelectrodes array recordings, and neuronal death, white matter rarefaction and microglia/macrophages density assessed in both hemispheres using imunohistochemistry. Brain and serum metabolites of the kynurenin pathway were measured using HPLC and mass fragmentography. At last, these same metabolites were measured in the patient’s serum, at the acute phase of stroke, to determine if they could predict PSCI 3 months later.

**Results:**

We found long-term spatial memory was impaired in diabetic mice 6 weeks after stroke induction. Synaptic plasticity was completely suppressed in both hippocampi along with increased neuronal death, white matter rarefaction in both striatum, and increased microglial/macrophage density in the ipsilateral hemisphere. Brain and serum quinolinic acid concentrations and quinolinic acid over kynurenic acid ratios were significantly increased compared to control, diabetic and non-diabetic ischemic mice, where PSCI was absent. These putative serum biomarkers were strongly correlated with degradation of long-term memory, neuronal death, microglia/macrophage infiltration and white matter rarefaction. Moreover, we identified these same serum biomarkers as potential predictors of PSCI in a pilot study of stroke patients.

**Conclusions:**

we have established and characterized a new model of PSCI, functionally and structurally, and we have shown that the QUIN/KYNA ratio could be used as a surrogate biomarker of PSCI, which may now be tested in large prospective studies of stroke patients.

**Supplementary Information:**

The online version contains supplementary material available at 10.1186/s13024-020-00421-4.

## Background

Stroke is a major cause of death, with 15 million deaths globally each year. It is the major cause of acquired disability and the second most frequent cause of dementia [1]**.** Up to 30% of stroke survivors develop dementia within 5 years. As life expectancy is prolonged [2], the sequels of stroke in survivors will generate increasing health costs. And yet, there is no biomarker to predict post-stroke dementia and no treatment is currently available to prevent or slow the progress of dementia after stroke.

Vascular cognitive impairment (VCI) encompasses all vascular contributions to cognitive impairment including stroke. It is a heterogeneous syndrome, including lesions of large and/or small brain vessels that lead to large infarcts or haemorrhages, lacunar infarcts, microbleeds and/or white matter lesions [3], and ultimately to memory degradation and executive dysfunction [4]. This concept emphasizes the fact that cardiovascular risk factors are involved, could be treated and that VCI could then be prevented [5, 6]. Recently, a group of experts proposed a consensus updated conceptualization of VCI in order to facilitate standardization in research [7], supported by imaging (i.e. diffuse white matter hyperintensities, ischemic strokes and lacunes, microbleeds …) [8]. They defined post-stroke cognitive impairment (PSCI) as occurring when cognitive function is degraded immediately or within 6 months of a stroke and does not recover.

The 5-year risk of dementia after stroke has been associated with a single vascular risk factor, diabetes, by recent data from the Oxford Vascular study group [9]. In the general population, diabetes prevalence may be as high as 10%. It significantly increases the risk for cardiovascular diseases [10] and has been associated with cognitive decline [11]. Systematic reviews have revealed an overall increased incidence of vascular dementia and Alzheimer’s disease in diabetic compared with non-diabetic patients [12]. However, diabetes is rarely integrated in animal models of post-stroke dementia and including this risk factor may well open new perspectives.

Pre-clinical models of post-stroke cognitive impairment (PSCI) are very diverse. Ischemic stroke has been induced by permanent or transient occlusion of the middle cerebral artery [13], by chronic hypoperfusion with arterial ligatures [14] or in genetic models such as muscarinic acetylcholine receptor M5 knockout mice [15]. Vascular risk factors have rarely been included in these studies [16–18]. Assessment of cognitive deficit and brain damage has also varied greatly between studies using Y maze or Morris water maze, blood-brain-barrier disruption or white matter rarefaction [19, 20].

This study therefore aimed to establish a reliable model of PSCI in diabetic mice. A permanent middle cerebral artery occlusion (pMCAo) was used to induce a small cortical infarct, comparable to those observed in the clinical setting. The model was validated with behavioral, electrophysiological, histological and serum biomarkers. Searching for a potential biomarker of PSCI, we focussed on tryptophan (TRP)-derived kynurenine (KYN) metabolism, since 1) post-stroke inflammation that develops early after stroke and persists over time may be a key factor in PSCI [21]; 2) inflammation activates indoleamine-2,3-dioxygenase (IDO), leading to an increased production of KYN and possibly TRP depletion, and thereby increasing the kynurenine/tryptophan (KYN/TRP) ratio, an index of IDO activity; 3) serum IDO activity has been correlated in 41 patients to cognitive impairment, 4 weeks after ischemic stroke [22]; 4) KYN metabolites, quinolinic acid (QUIN) [23, 24] and kynurenic acid (KYNA) [25] are known to induce synaptic plasticity. We therefore used this stroke-diabetes mouse model to ask whether serum metabolites of the kynurenine pathway (KP) could be useful biomarkers, correlated with defined indices of PSCI. We then measured serum metabolites of the kynurenine pathway in post-stroke patients, to investigate if they could be good predictors of post-stroke cognitive decline.

## Methods

All experiments and surgical procedures were performed according to European Community Directive (2010/63/EU) and the French National guidelines for the care and use of laboratory animals. The study was specifically approved by the Local Ethics Committee in Animal Experimentation and by the French ministry of Higher Education for Research and Innovation (APAFIS#4100-2015111714376561v6).

### Experimental design (Fig. 1)

Focal cerebral ischemia was induced, in diabetic and non-diabetic mice, by permanent middle cerebral artery thermocoagulation (pMCAo) on day 0 (D0). Sensorimotor examination was conducted 1 day before surgery (D-1), then at D1, D3, D7, D14 and D21. An index of “anxiety” was derived from the open field test at D28, and the marble burying test at D29, and an index of “depression” was obtained using the splash test at D29. Spatial memory was evaluated between D30 and D40 with the Barnes maze test. These multimodal behavioural analyses were made sequentially to avoid bias. First, we established that if stroke diabetic mice had a greater sensorimotor deficit than stroke non-diabetic mice, the sensorimotor score was normalized by D21, so performance on the Barnes maze would not be affected by deficit. For the same reason, because anxiety and depression are two common manifestations that develop after stroke [26], and might interfere with the Barnes maze test interpretation, open field, the marble burying test and the splash test were performed at D28 and D29.

**Fig. 1:**
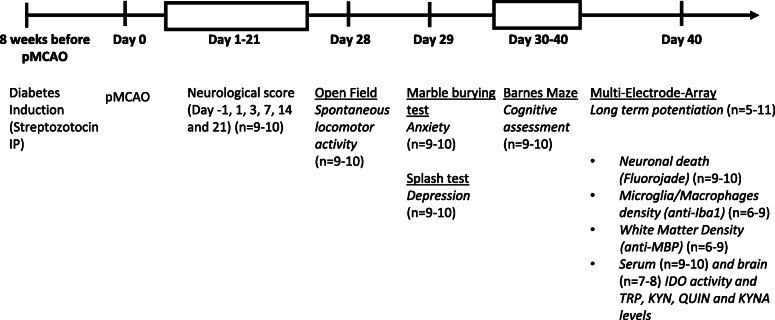
Timeline of experimental protocol. pMCAo: permanent middle cerebral artery occlusion; IP: intraperitoneal; n: number of mice/group. Long term potentiation, neuronal death and microglia/macrophage densities were studied in both hippocampus; white matter density and microglia/macrophages density were studied in both striatums; microglia/macrophage density was studied in the peri-infarct and contralateral homonymous area. Indoleamine 2, 3 dioxygenase (IDO) activity that converts Tryptophan (TRP) to Kynurenine (KYN), and kynurenine metabolites, quinolinic acid (QUIN) and kynurenic acid (KYNA) were measured in serum and brain at D40. Four groups were evaluated for each time point: control mice (C), diabetic mice (D), pMCAo mice (mice subjected to permanent middle cerebral artery occlusion without diabetes) and pMCAo + D (mice subjected to permanent middle cerebral artery occlusion with diabetes); n is the number of mice/group

Long-term potentiation (LTP) at hippocampal synapses was measured at D40. Histology, immunohistochemistry, and biochemical studies were made on brain tissue and blood sampled at D40. The following groups were investigated: control mice without diabetes and without pMCAo (C); diabetic mice without pMCAo (D); non-diabetic mice subjected to focal cerebral ischemia (pMCAo), and diabetic mice subjected to focal cerebral ischemia (pMCAo+D). All scorings and analyses were made by investigators blind to animal status in these groups. Behavioural, histological, immunohistochemical, and serum biochemical tests were performed in the same animals, whereas LTP studies and brain biochemical tests were performed in a subset of mice.

### Diabetes induction

Six-week-old male C57BL/6 J mice (Janvier, Le Genest Saint-Isle, France) were divided into two groups: one received five consecutive daily intraperitoneal (IP) injections of Streptozotocine (STZ, 60 mg/kg in 100 μL of citrate buffer) to induce diabetes; the other group received a sham injection of citrate buffer. Glycemia was assessed weekly for 8 weeks. Mice with sustained hyperglycemia (> 300 mg/dL) were considered as diabetic (90% of treated mice). Mice were housed in a 12-h light-dark cycle, with free access to food and water.

### Permanent middle cerebral artery occlusion (pMCAo)

Fourteen-week-old male C57BL/6 J mice were anesthetized with isoflurane (1.5 to 1.8% in O_2_) and body temperature was continuously monitored and maintained at 37 ± 0.5 °C using a heating blanket (Homeothermic Blanket Control Unit; Harvard Apparatus Limited, UK). Mice were subjected to pMCAo by thermocoagulation of the middle cerebral artery, as described elsewhere [27]. D0 refers to the day of pMCAo.

### Sensorimotor assessment

Mice were trained on sensorimotor procedures on the day before pMCAo to avoid stress bias. Periodically after pMCAo (D1, D3, D7, D14 and D21), mice were assessed on five tests: neurological score, grip and string test, beam walking and pole test. Results from these tests are given as neurological score. The maximal score value for this index was 19 with lower scores indicating a more severe deficit [27, 28].

### Cognitive assessment

Mice were housed in the behaviour room so that they could be accustomed to the environment where they were subjected to a 12-h night-day cycle with food and water ad libitum. Before testing spatial memory at D40 with the Barnes maze (BM), the absence of residual sensorimotor deficit was verified by determining the global neurological score at D21. Impulsivity and anxiety-related behaviour were assessed at D28 and D29 with the open field and marble burying test. The splash test was performed at D29 to assess anhedonia, a symptom of depression.

#### Anxiety

Anxious mice tend to freeze, a behaviour inconsistent with accurate results in the Barnes maze test. We used the open field and marble burying tests to detect anxiety. In the open field test, mice were placed in a chamber (PVC; 50x50x40cm; TSE Systems GmbH, Bad Homburg, Germany). Spontaneous activity was measured as the total distance travelled (cm) from images obtained with an infrared video camera by EthoVision XT 11.5 Tracking Software (Noldus Wageningen, Holland). In the marble burying test, twenty black glass marbles were arranged in a 4 × 5 equidistant grid pattern on top of sawdust in a cage. Animals entered the cage for 30 min. After 30 min the number of buried marbles (including those only 2/3 covered) was counted. The number of buried marbles was assumed to be negatively correlated with anxiety [29].

#### Depression

In the splash test, used to assess depression, an animal was placed in a cage for 2 min and a 10% sucrose solution was vaporized onto its dorsal coat. The total duration (seconds) of grooming, licking or scratching to remove the sucrose, was determined over 5 min [30] [31]**.**

#### Spatial learning

The Barnes maze was used to test spatial learning and memory as we previously described [21]. Briefly, 20 holes are spaced around the maze, one-meter diameter brightly illuminated, and the mouse must find an “escape box” hidden under one of the holes. The first 4 days (1st to 4th day) consisted of the learning phase. After 2 days of rest, the mice were placed back on the platform (7th day) and had to remember the location of the escape box (retention phase). Finally, the next day, the escape box was moved to a different location, and the ability of the mouse to learn and remember the new location was measured for 4 days (8th, 9th, 10th and 11th day) (reversal phase). Mouse behaviour was filmed and the time required to find the escape box measured (latency to escape in seconds).

### Electrophysiological assessment

At 6 weeks after cerebral artery occlusion, we evaluated long-term potentiation (LTP) of synaptic efficacy induced by high-frequency stimulation [32], in hippocampal slices.

#### Slice preparation

14-week-old mice were anesthetized with IP injection of ketamine and xylaxine (20 and 10% respectively) and sacrificed by decapitation after 2–3 min intra-cardiac perfusion of a 4 °C sucrose-based artificial cerebrospinal fluid (aCSF). The aCSF contained: 250 mM sucrose, 3 mM KCl, 25 mM NaHCO_3_, 10 mM D-Glucose, 1 mM CaCl_2_, 10 mM MgCl_2_ (in mM). It was warmed to 34 °C and equilibrated with 5% CO_2_ in 95% O_2_. The brain was carefully removed and 400 μm thick slices were cut in a vibratome (Leica VT1200S, Germany) in the oxygenated sucrose-based aCSF. Slices containing the hippocampus were stored in an interface chamber (Brain Slice Chamber 2, Scientific Systems Design, USA) at 34 °C and perfused at 2 ml/min for at least 1 h with an aCSF containing (in mM): 124 NaCl, 3 KCl, 26 NaHCO_3_, 10 D-Glucose, 1.6 CaCl_2_, 1.3 MgCl_2_, bubbled with 5% CO_2_ in 95% O_2_.

#### Multi-electrode array (MEA) recordings

Hippocampal slices were transferred from the interface chamber to planar MEA petri dishes (200–30 indium-tin oxide electrodes of 30 μm diameter at 200 μm separation in a 12 × 12 matrix, with a reference electrode; Multichannel Systems, Germany). Slices were perfused with aCSF at 2 ml/min with a platinum anchor used to prevent movement. Electrical signals were acquired at 10 kHz with a MEA2100–120 system (MultiChannel Systems) and software (MC_Rack, MultiChannel Systems) [33]. Schaffer collateral fibres were stimulated with biphasic 100 μs voltage pulses delivered in monopolar mode by one of the array electrodes. Stimulus strength and frequency were controlled by MC Rack software. Stimuli were adjusted to induce a field Excitatory Post Synaptic Potential (fEPSP) in the CA1 area [34]. After recording stable fEPSP responses to Schaffer collateral stimulation at interval 10 s for at least 10 min, tetanic, high-frequency stimulation was applied (5 trains of 100 stimuli at 100 Hz). The interval between stimuli was returned to 10 s, and fEPSP amplitude was monitored for 45 min. fEPSP amplitude before and 45 min after tetanic stimulation, were compared for mice of the four groups: with or without diabetes, with or without stroke. LTP was measured from the ipsi-lateral and contralateral hippocampus with results expressed as the % change from baseline fEPSP values.

### Biochemical assays

Tryptophan (TRP) is mainly utilized for protein synthesis (> 90%), but a small fraction is converted into serotonin and kynurenine (KYN) [35]. KYN is in turn metabolized into kynurenic acid (KYNA) or quinolinic acid (QUIN). TRP, KYN, and KYNA were measured using HPLC, whereas QUIN was measured by mass fragmentography as previously described [36]. These metabolites were expressed in μM (TRP, KYN), and nM (KYNA, QUIN). The in vivo Indoleamine 2,3 Dioxygenase (IDO) activity was estimated by the product to substrate (KYN/TRP) ratio and expressed as a percentage (%).

Brains were weighed and sonicated at 4 °C until complete homogenization in an ice-cold ascorbate/HClO_4_10^−2^M solution (dilution 1/10, w/v). The samples were then centrifuged at 12,000 g for 5 min at 4 °C. The supernatant was removed and placed in a Costar Spin-X tube filter and then centrifuged at 12,000 g for 5 min at 4 °C. The brain samples were then diluted to 1/10 in a 0.06 mM ascorbate solution. Serum (100 μL) was mixed with 10 μL 0.1% (v/v) aqueous formic acid (FA) and 370 μL ice-cold acetone–methanol (1:1, v/v) containing 10 μL internal standard (e.g. 25 nM d3-QUIN for quinolinic acid determination). It was allowed to rest for 15 min at − 20 °C, vortexed for 60s to support protein precipitation, and incubated for another 15 min at − 20 °C. The supernatant was obtained via centrifugation of the mixture for 15 min at 12,000 g at 4 °C.

For TRP, KYN and KYNA analysis a high-performance liquid chromatography technique based on an ESA Coulochem III detector was used. The liquid phase (pH = 3.0) consisted of a solution of 75 mM NaH_2_PO_4_, 25 μM EDTA, 1.7 mM octanesulfonic acid and triethylamine in acetonitrile water (100 μl/L). For quinolinic acid determination 400 μL supernatant were transferred to a new tube, centrifuged for 15 s, and split into two equal parts. After concentration under vacuum (Savant SC 110 A Speed Vac Plus, Savant, USA), half of the sample was treated with 70 μL derivatizing reagent (n-butanol-acetylchloride, 9:1, v/v) and incubated for 1 h at 60 °C. The mixture was dried under nitrogen before reconstitution. Both parts of the sample were dissolved in 100 μL starting eluent (10% methanol containing 0.1% (v/v) FA in 0.1% (v/v) aqueous FA), vortexed, centrifuged, and combined. Finally, 20 μL of the sample was injected into the UHPLC–MS/MS system (ACQUITY I-Class UPLCTM liquid chromatography system, Waters, Manchester, UK).

### Histological analysis

On the day of sacrifice (D40), mice were transcardially perfused with heparinized saline, followed by 4% paraformaldehyde (PFA) in 0.1 M phosphate buffer, pH 7.4. Brains were removed, post-fixed overnight in PFA and cryoprotected in 20% sucrose. Thirty μm thick floating coronal sections were incubated with a primary antibody overnight at 4 °C. Immunohistochemistry was performed with anti-Iba 1 (Ionized calcium binding adaptor molecule 1, 1:400, Wako, Japan), anti-MBP (Myelin Basic Protein, 1:1600. Millipore, USA) antibodies to detect microglia/macrophages and white matter, respectively. Secondary antibodies used Alexa-Fluor 488 as fluorophore (1:400, Molecular Probes, Eugene, OR). Fluorojade staining (Millipore, USA) was used to estimate neuronal degeneration.

### Morphological analysis

***Neuronal death*** was assessed in both whole hippocampi by counting the number of fluorojade+ cells at − 1.28 mm and − 2.12 mm relative to bregma. ***Microglia/macrophages*** cell density was assessed by calculating the Iba1 area in two randomly chosen regions of interest (20X) in both hippocampi, both striata, in the peri-infarct area and in the homonymous contralateral area. ***White matter*** density was evaluated by calculating the MBP+ area in both striata at + 0.74 mm relative to bregma. These cells densities were expressed as contrast density using NIH ImageJ software (arbitrary units)***.***

### Patients

Blood samples of patients admitted in the stroke unit of our hospital for thrombolysis and/or thrombectomy are systematically stored at the acute phase (BioBank N° BB-0033-00064). We measured and compared tryptophan-derived kynurenine metabolites from blood samples of consecutive patients admitted in the stroke unit and who had had a neuropsychological assessment 3 months or more after their stroke. Patients were then classified according to their cognitive status (Mini Mental State examination, Montreal Cognitive assessment, Wais-IV, Frontal Assessment Battery, trail making test B, trail B-trail A, Rey Figure, Stroop Victoria). As a retrospective evaluation, the battery of tests was not standardized. Patients were classified as having no cognitive decline (C-) or with global cognitive decline/executive dyfunction (C+). In addition, gender, age, infarct location, vascular risk factors, stroke etiology according to TOAST classification [37] was collected.

This study was approved by by our Institutional Review Board (IRB00003888; number 20–695), and therefore, has been performed in accordance with the ethical standards laid down in 1964 declaration of Helsinki and its later amendments.

### Statistical analysis

Prism 6 software (GraphPad, San Diego, CA) was used for statistical analyses. Data were expressed as mean ± SD. The Shapiro-Wilk normality test was used to test whether data might conform to a Gaussian distribution. Data comparisons between groups were performed using the Wilcoxon test for pre- and post-tetanic fEPSPs in LTP experiments; t-test or Mann & Whitney test was used to compare serum metabolites in the two groups of patients and effect size calculated. Fishers’exact test was used for comparison of their demographic and clinical data. Two-way ANOVA tests for repeated measures were used to analyse sensorimotor data and learning or reversal phases from Barnes maze tests. All other evaluations were made using 2-way ANOVA tests with a *post-hoc* Tukey test. Grubb’s test was used to identify outliers. Statistical significance was set at *P <* 0.05. Spearman correlations were used to assess possible relations between the kynurenine pathway and functional assessments or immunohistochemical data, across the groups and within each group.

## Results

### Stroke diabetic mice present a more severe sensorimotor deficit that recovers by D21

**Fig. 2:**
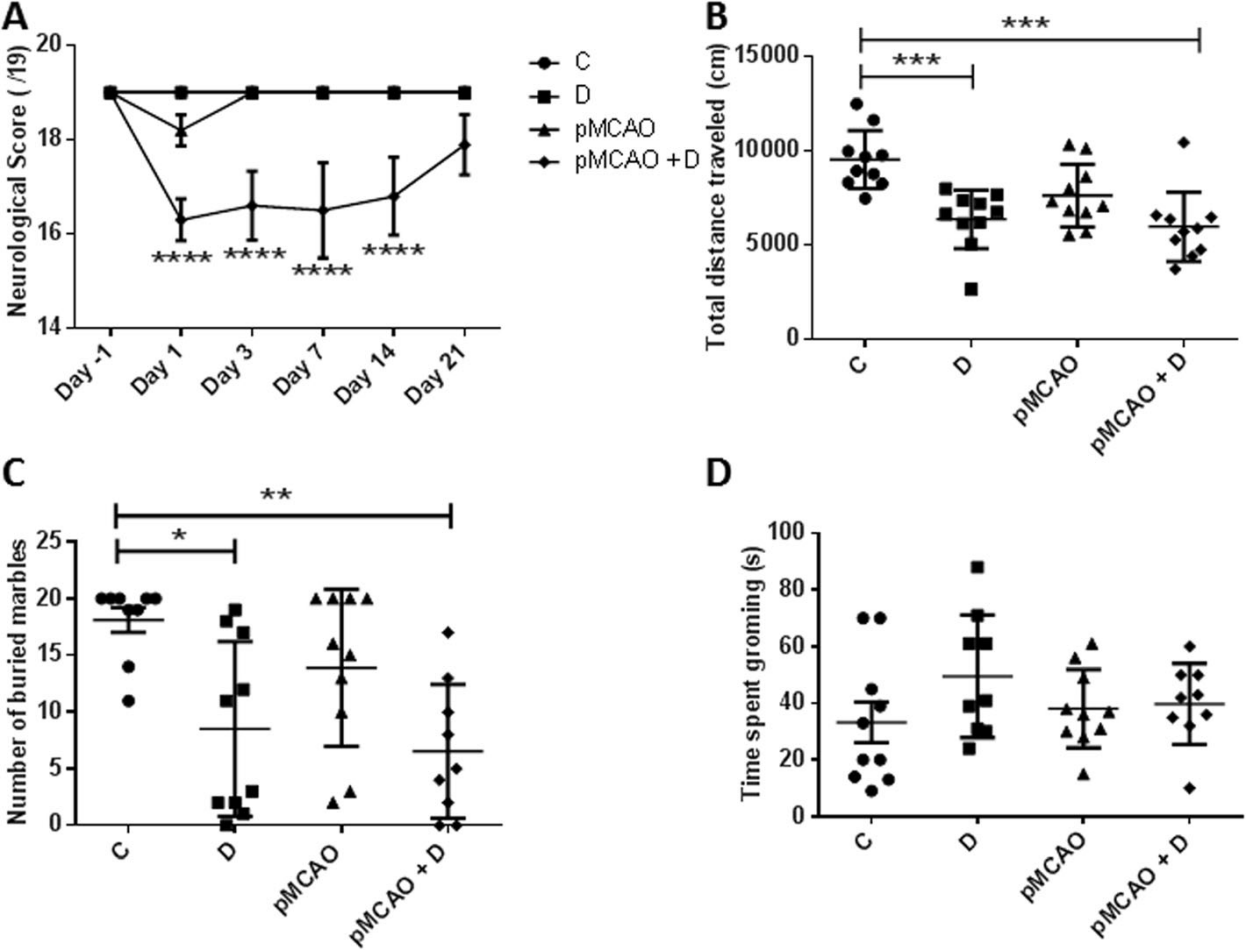
Functional evaluations were made in C, D, pMCAo and pMCAo+D mice. **a** From D1 to D14, the pMCAo + D group had significantly lower neurological scores than C, D and pMCAo mice) (*P <* 0.0001). By D21, pMCAo + D mice had completely recovered and no significant differences between groups remained. **b** In the open field test, at D28, the total distance travelled by pMCAo+D mice was significantly less than that of C mice (*P <* 0.001) and also less for D mice than C mice (*P <* 0.001). **c** In the marble burying test, at D29, significantly fewer marbles were buried by pMCAo+D mice than by C mice (*P <* 0.01) and by D mice compared to C mice (*P <* 0.05). **d** There were no significant differences between the 4 groups in the splash test at D29. *P < 0.05, **P < 0.01, ***P < 0.001, ****P < 0.0001

The neurological score of pMCAo+D mice was significantly lower on the day after arterial occlusion than on the day before occlusion (*P <* 0.0001). Neurological scores for this group of mice were consistently worse than those of C, D and pMCAo animals at D1, 3, 7 and 14 (*P <* 0.0001). At day 21, sensorimotor deficit had recovered in pMCAo mice with no significant differences in neurological score to the other animal groups (*n* = 9–10) (time effect, *P <* 0.0001; group effect, *P <* 0.0001; time*group, *P <* 0.0001) (Fig. 2a and Table 1).

**Table 1:** Sensorimotor assessment. Control mice (C), diabetic mice (D), mice subjected to permanent middle cerebral artery occlusion (pMCAo) and diabetic mice subjected to permanent middle cerebral artery occlusion (pMCAo+D)

Day	C (***n*** = 10)	D (***n*** = 9)	pMCAo (***n*** = 10)	pMCAo + D (***n*** = 10)
**D-1**	19.0 ± 0.0	19.0 ± 0.0	19.0 ± 0.0	19.0 ± 0.0
**D1**	19.0 ± 0.0	19.0 ± 0.0	18.2 ± 1.0	13.6 ± 2.5
**D3**	19.0 ± 0.0	19.0 ± 0.0	19.0 ± 0.0	13.1 ± 4.4
**D7**	19.0 ± 0.0	19.0 ± 0.0	19.0 ± 0.0	13.8 ± 4.0
**D14**	19.0 ± 0.0	19.0 ± 0.0	19.0 ± 0.0	15.2 ± 1.7
**D21**	19.0 ± 0.0	19.0 ± 0.0	19.0 ± 0.0	17.9 ± 2.0

### Diabetic mice with or without stroke show increased anxiety features; no depression is evidenced in any group (Fig. 2b-d)

Having shown that sensorimotor function had recovered at D21 after cerebral artery occlusion in pMCAo+D mice, we asked whether anxiety or depression could interfere with tests of spatial memory (*n* = 9–10). Both D and pMCAo+D mice were more anxious according to the open field and marble burying task. At open field, diabetic animals with or without stroke, covered lower distances than control mice (*P <* 0.001) but were similar to pMCAo mice (diabetes effect, *P <* 0.0001; stroke effect, *P =* 0.03; diabetes*stroke effect, *P =* 0.16) (Fig. 2b). Similarly, they buried significantly fewer marbles than control mice (pMCAo+D, *P <* 0.01; D, *P <* 0.05) but differences with pMCAo mice were not significant (diabetes effect, *P =* 0.0002; stroke effect, *P =* 0.14; diabetes*stroke effect, *P =* 0.58) (Fig. 2c). Since sensorimotor function had normalized at this time, residual deficits could not be involved. Data from the splash test did not indicate mice were in a depressive state. There were no significant differences in grooming time between animal groups (diabetes effect, *P =* 0.15; stroke effect, *P =* 0.68; diabetes*stroke effect, *P =* 0.23) (Fig. 2d).

### Only pMCAo+D mice present a cognitive decline (Fig. 3 and Table 2)

**Fig. 3:**
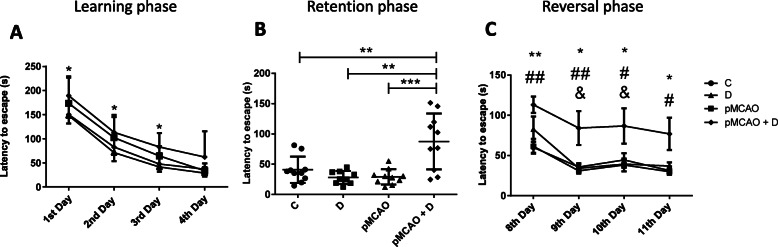
Spatial memory evaluated by the Barnes maze between D30 and D40. **a** During the learning phase, the latency to escape of pMCAo+D mice was significantly longer on the first three days than that for the other three groups (*P <* 0.05) but was no longer significantly different on the 4th day. **b** During the retention phase, pMCAo+D mice showed significantly longer latencies to escape than all other groups: C (*P <* 0.01), D (*P <* 0.01) and pMCAo mice (*p* < 0.001). **c** During the 4 days of the reversal phase, pMCAo+D mice showed significantly longer latencies to escape than animals of all other groups (*P <* 0.05). Overall, pMCAo+D mice showed spatial memory deficits and impaired plasticity especially during the retention and reversal phases of the maze test. For the learning phase *P < 0.05 between pMCAo+D and pMCAo mice; for the retention phase, **P < 0.01, ***P < 0.001; for the reversal phase, *P < 0.05 and **P < 0.01, between pMCAo+D and C mice; # *P <* 0.05 and ##*P <* 0.01 between pMCAo+D and pMCAo mice; & *P <* 0.05, between pMCAO+D and D mice

Animals from all four groups learned the Barnes maze task, with significant decreases in the latency to escape between the 1st and the 4th training day (*P <* 0.0001). pMCAo+D animals took longer than non-diabetic pMCAo mice on the 1st day, the 2nd day and the 3rd day (*P <* 0.05), but no deficit remained on the 4th day (Fig. 3a). Spatial memory performance was not affected at this time point (subject effect, *P* < 0.0001; time effect, *P =* 0.086; group effect, *P =* 0.006; interaction effect, *P =* 0.9474).

**Table 2:** Barnes maze assessment. Control mice (C), diabetic mice (D), mice subjected to permanent middle cerebral artery occlusion (pMCAo) and diabetic mice subjected to permanent middle cerebral artery occlusion (pMCAo + D)

Days	C (***n*** = 10)	D (***n*** = 9)	pMCAo (***n*** = 10)	pMCAo + D (***n*** = 10)
**1st**	162.9 ± 51.5	173.4 ± 55.7	148.2 ± 25.39	189.4 ± 37.7
**2nd**	81.1 ± 31.5	102.5 ± 42.5	71.3 ± 40.65	113.5 ± 35.8
**3rd**	47.9 ± 18.1	41.7 ± 28.8	64.5 ± 20.10	83.1 ± 28.7
**4th**	41.4 ± 23.5	32.9 ± 11.4	33.9 ± 15.15	62.1 ± 53.7
**7th**	40.7 ± 21.8	28.0 ± 10.7	28.9 ± 12.6	87.6 ± 46.2
**8th**	60.2 ± 21.8	82.9 ± 47.0	61.5 ± 29.1	113.2 ± 32.6
**9th**	36.7 ± 15.2	34.1 ± 15.2	31.0 ± 38.6	84.1 ± 67.0
**10th**	44.6 ± 26.6	39.3 ± 13.9	38.3 ± 25.4	86.8 ± 69.7
**11th**	31.3 ± 16.6	36.9 ± 13.6	29.9 ± 9.5	77.0 ± 63.4

On the 7th day of the retention phase, pMCAo+D mice took longer times to find the escape box than the three other animal groups: C (*P <* 0.01), D (*P <* 0.01) and pMCAo mice (*P <* 0.001; *n* = 9–10) (Fig. 3b). ANOVA analysis showed the diabetic*stroke effect was most significant (diabetic effect, *P =* 0.0122; stroke effect, *P =* 0.0096; diabetic*stroke effect, *P =* 0.0002).

pMCAo+D mice also performed poorly during the reversal phase. Latency for these animals to find the escape box was significantly longer on the 8-11th days than for C (*P <* 0.01 for the 8th day, *P <* 0.05 for 9-11th days), D (*P <* 0.05 for the 9th and 10th day) or pMCAo mice (*P <* 0.01 for the 8th and 9th day, and *P <* 0.05 for the 10th and 11th day) (Fig. 3c). ANOVA analysis suggests pMCAo+D mice (*n* = 9–10) do not adapt efficiently to novel instructions (subject effect, P < 0.0001; time effect, *P <* 0.0001; group effect, *P =* 0.0025; interaction, *P =* 0.8105).

The degraded performance of pMCAo+D mice on the spatial memory task cannot be explained by an enhanced anxiety. Diabetic mice, with or without cerebral ischemia, had higher scores on anxiety tests but only pMCAo+D animals were deficient in the spatial memory test.

### Long-term potentiation is impaired in mice subject to cerebral ischemia

**Fig. 4:**
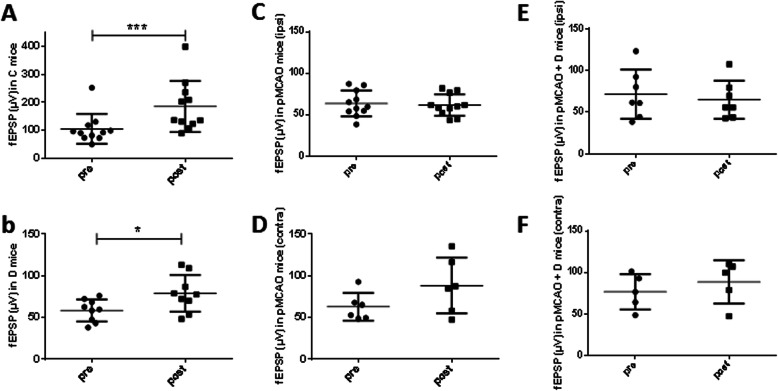
Effects of pMCAo and diabetes on long-term potentiation (LTP) of CA1 field Excitatory Post-Synaptic Potentials (fEPSP) induced in hippocampal slices by tetanic stimulation of Schaffer collaterals. Graphs show fEPSP amplitudes (μV) before (pre) and after (post) tetanic stimulation in (**a**) control, (**b**) diabetic, (**c**, **d**) pMCAo and (**e**, **f**) pMCAo+D mice. LTP was assessed for both pMCAo and pMCAo+D mice in hippocampus ipsilateral (**c**, **e**) or contralateral (**d**, **f**) to the infarct. Significant synaptic LTP was detected in slices from control (*P <* 0.001) and diabetic mice (*P <* 0.05). LTP was not induced in either ipsi- or contralateral slices from both pMCAo and pMCAo+D mice. fEPSP, field excitatory post synaptic potential. *P < 0.05, ***P < 0.001

**Fig. 5:**
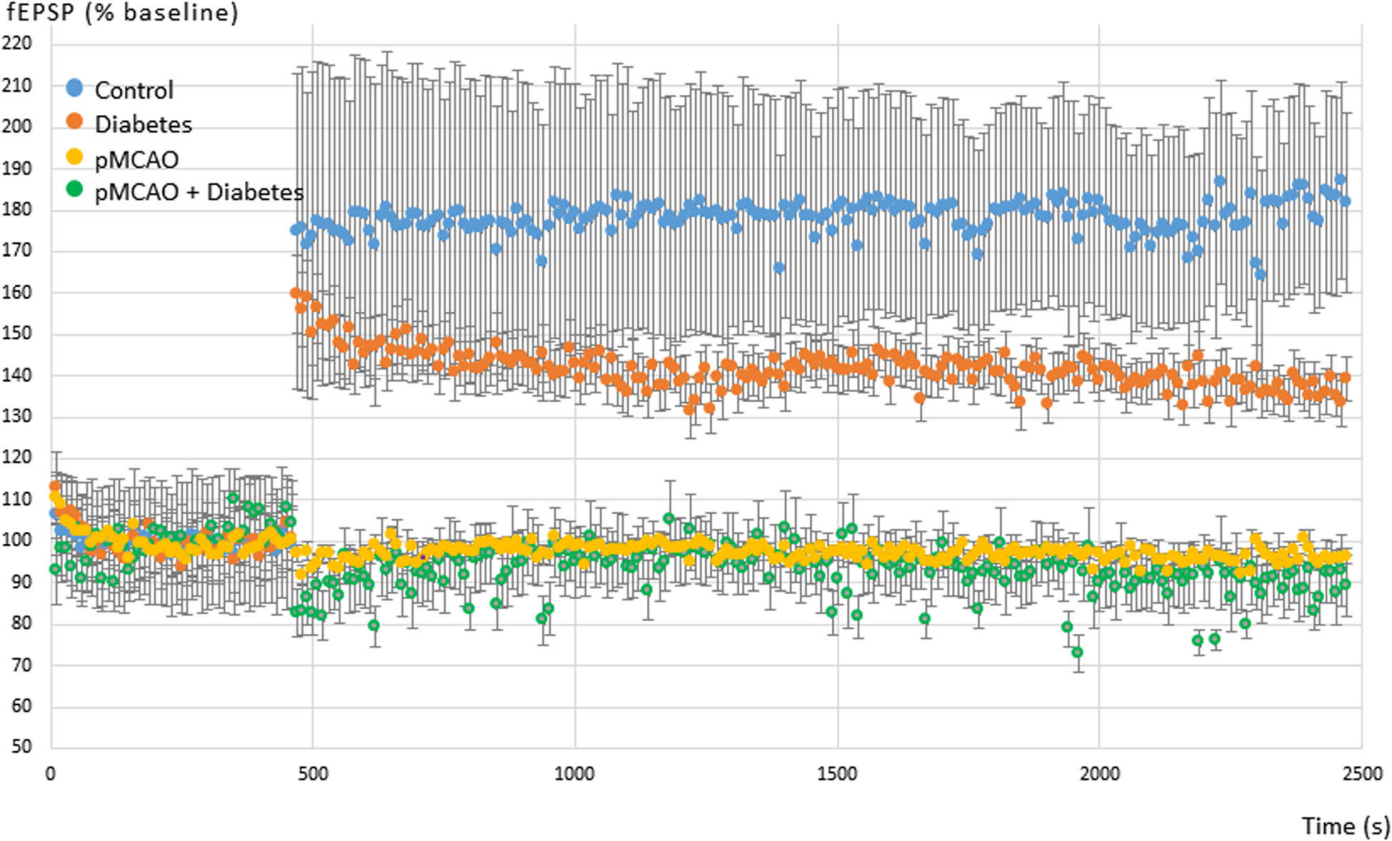
Mean and SEM of fEPSP amplitudes in the CA1 region of hippocampal slices from the 4 different groups of mice before and after tetanic stimulation of Schaffer collaterals. Amplitudes are normalized to 100% for the control period from 0 to 480 s when the tetanic stimulation was delivered. Data are shown from control mice (blue), diabetic mice (orange), pMCAo mice (yellow) and pMCAo diabetic mice (green). LTP was not induced in pMCAo animals, with or without diabetes

After the LTP induction procedure, fEPSPs were significantly potentiated in both C (185.5 ± 91.3 μV vs. 104.9 ± 53.6 μV) (*n* = 11) (*P <* 0.001) (Fig. 4a) and D mice (78.8 ± 22.0 μV vs. 58.5 ± 13.2 μV; *n* = 9; *P =* 0.05) (Fig. 4b). In contrast no significant potentiation was detected in pMCAo mice (ipsilateral hippocampus, 61.7 ± 12.9 μV vs. 63.6 ± 15.5 μV, *n* = 11; contralateral hippocampus, 88.2 ± 33.5 μV vs. 62.8 ± 16.7 μV; *n* = 6) (Fig. 4c, d) or in pMCAo+D (ipsilateral, 65.0 ± 22.9 μV vs. 71.7 ± 29.5 μV, *n* = 7; contralateral hippocampus, 88.6 ± 26.0 μV vs. 76.7 ± 21.2 μV; *n* = 5) (Fig. 4e, f). Thus stroke apparently suppressed LTP in both ipsilateral and contralateral hippocampus. We note that mean basal fEPSP amplitudes differed: 178.7 ± 42.6 μV in C mice, 143.1 ± 64.6 μV in D mice, 100.1 ± 25.4 μV in pMCAo animals and 94.6 ± 20.8 μV in pMCAo+D animals. For comparison between groups, fEPSP amplitudes were normalized to a percentage of the baseline (Fig. 5).

### Serum and brain changes in kynurenine pathways are found specifically in pMCAo+D mice

**Fig. 6:**
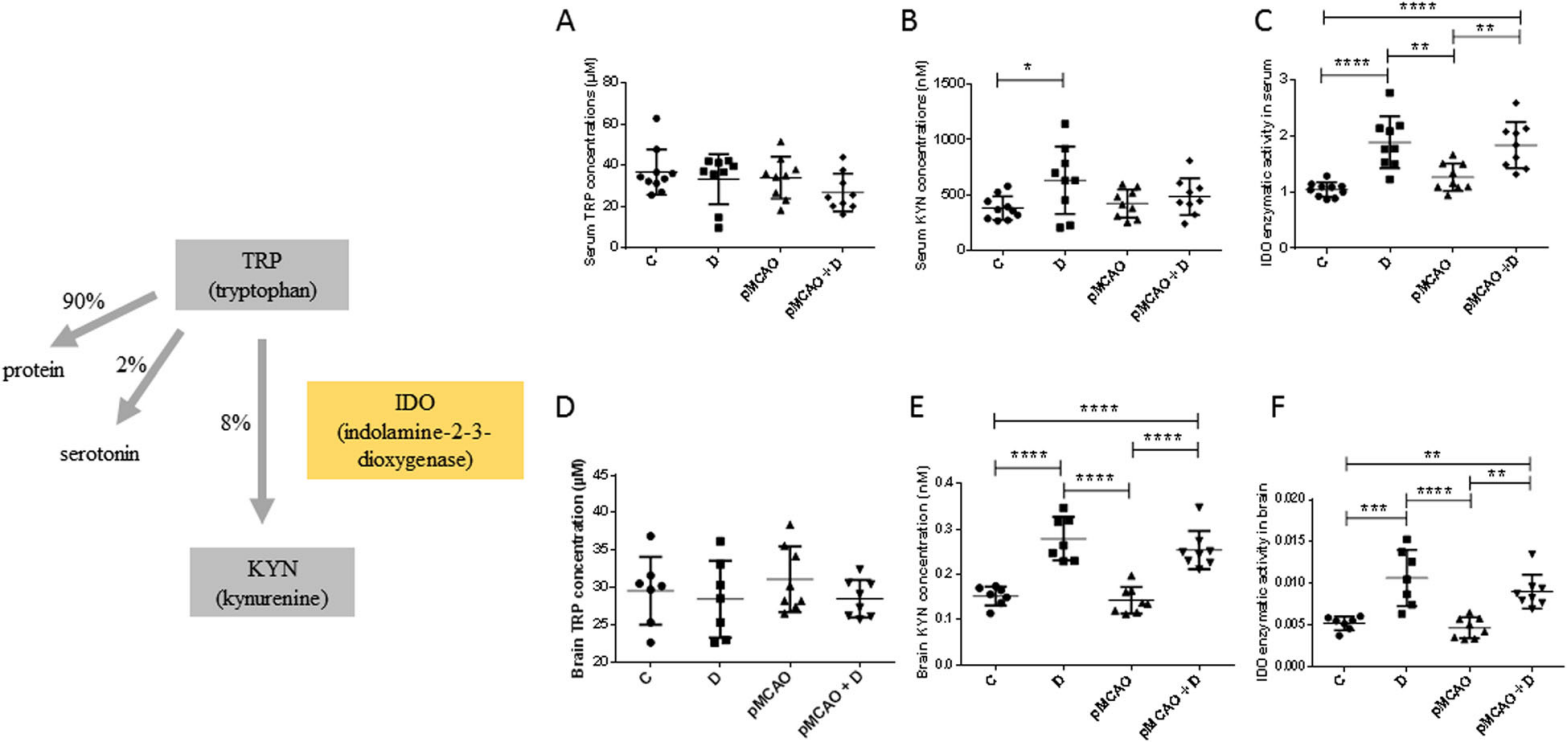
Serum and brain changes of the tryptophan (TRP) and kynurenine (KYN) concentrations and indoleamine 2, 3-dioxygenase (IDO) activity in pMCAo+D mice. These elements of the tryptophan/kynurenine pathway are shown at the left. **a-f** show changes in concentrations or activity of these molecules in the C, D, pMCAo and pMCAo+D mice, in serum (**a**, **b**, **c**) and brain (**d**, **e**, **f**). **a** Serum TRP levels did not differ between the four groups. **b** Serum KYN levels were significantly higher in D than in C mice (*P <* 0.05). **c** Serum IDO activity was significantly higher in pMCAo+D animals than in pMCAo mice (*P <* 0.01) or C mice (*P <* 0.0001). It was higher in D mice than in pMCAo (*P <* 0.01) or C mice (*P <* 0.0001). **d** Brain TRP levels did not differ between the four groups. **e** Brain KYN levels were significantly higher in pMCAo+D animals than in pMCAo or C mice (*P <* 0.0001). They were higher in D mice than in pMCAo or C mice (*P <* 0.0001). **f** Brain IDO activity was significantly higher in pMCAo+D animals than in pMCAo mice (*P <* 0.01) or C mice (*P <* 0.01). It was higher in D mice than in pMCAo (*P <* 0.0001) or C mice (*P <* 0.001) (*P < 0.05, **P < 0.01, ***P < 0.001, ****P < 0.0001)

There were no significant differences in serum TRP levels in different groups of animals (C mice, 36.7 ± 11.0 μM; D mice, 33.2 ± 12.2 μM; pMCAo 33.9 ± 10.2 μM; pMACo+D mice, 26.8 ± 9.2 μM) (*n* = 9–10) (diabetes effect, *P =* 0.14; stroke effect, *P =* 0.20; diabetes*stroke effect, *P =* 0.60) (Fig. 6a). No significant differences in brain TRP levels in different groups of animals were found either (*n* = 7–8) (diabetes effect, *P =* 0.24; stroke effect, *P =* 0.61; diabetes*stroke effect, *P =* 0.64) (Fig. 6d). Serum KYN levels were significantly elevated in D mice (632 ± 307 nM) compared to C mice (379 ± 107 nM) (*P <* 0.05) (*n* = 9–10) (diabetes effect, *P =* 0.02; stroke effect, *P =* 0.40; diabetes*stroke effect, *P =* 0.14) (Fig. 6b). Brain KYN levels were significantly higher in pMCAo+D animals than in pMCAo or C mice (*P <* 0.0001). They were higher in D mice than in pMCAo or C mice (*P <* 0.0001) (*n* = 7–8) (diabetes effect, *P <* 0.0001; stroke effect, *P =* 0.21; diabetes*stroke effect, *P =* 0.57) (Fig. 6e). Serum activity of IDO, the rate limiting enzyme that converts TRP to KYN, was significantly higher in pMCAo+D mice (1.83 ± 0.42%) than in pMCAo (1.26 ± 0.25%; *P <* 0.01) or C mice (1.04 ± 0.13%; *P <* 0.0001). IDO activity was also higher in D mice (1.88 ± 0.46%) than in pMCAo (*P <* 0.01) or control mice (*P <* 0.0001) (*n* = 9–10) (Fig. 6c). Diabetes alone increased serum IDO activity (diabetes effect, *P <* 0.0001; stroke effect, *P =* 0.45; diabetes*stroke effect, *P =* 0.23). Brain IDO activity was significantly higher in pMCAo+D animals than in pMCAo mice (*P <* 0.01) or C mice (*P <* 0.01). It was higher in D mice than in pMCAo (*P <* 0.0001) or C mice (*P <* 0.001) (*n* = 7–8) (diabetes effect, *P <* 0.0001; stroke effect, *P =* 0.16; diabetes*stroke effect, *P =* 0.80) (Fig. 6f).

**Fig. 7:**
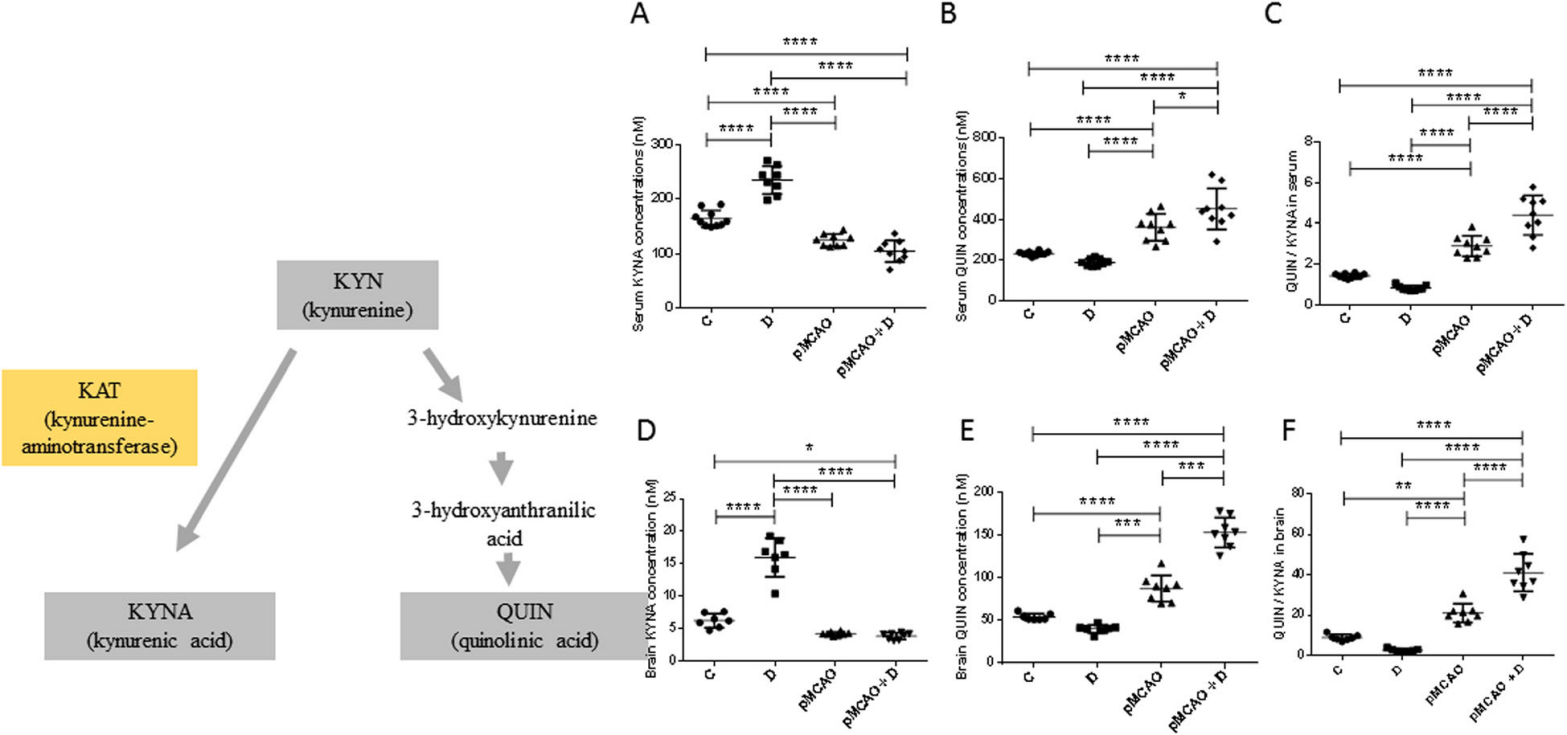
Serum and brain changes of the kynurenic acid (KYNA) and quinolinic acid (QUIN) concentrations and QUIN/KYNA ratio in pMCAo+D mice. These elements of the tryptophan/kynurenine pathway are shown at the left. **a**-**f** show changes in concentrations or activity of these molecules in the C, D, pMCAo and pMCAo+D mice, in serum (**a**, **b**, **c**) and brain (**d**, **e**, **f**). **a** Serum KYNA concentrations were lower in pMCAo+D mice than in D or C mice (*P <* 0.0001). KYNA levels were lower in pMCAo mice than in D and C mice (*P <* 0.0001) and lower in C mice than in D mice (*P <* 0.0001). **b** Serum QUIN levels were significantly higher in pMCAo+D than in C mice (*P <* 0.0001), D (*P <* 0.0001) or in pMCAo mice (*P <* 0.05). They were higher in pMCAo mice than in C or D mice (*P <* 0.0001). **c** The serum QUIN/KYNA ratio was higher in pMCAo+D mice than in all other groups (*P <* 0.0001). The ratio was significantly higher in pMCAo mice than in D or C mice (*P <* 0.0001). **d** Brain KYNA concentrations were lower in pMCAo+D mice than in D (*P <* 0.0001) or in C mice (*P <* 0.05). Brain KYNA levels were lower in pMCAo mice than in D (*P <* 0.0001) and lower in C mice than in D mice (*P <* 0.0001) (*P < 0.05, **P < 0.01, ***P < 0.001, ****P < 0.0001). **e** Brain QUIN levels were significantly higher in pMCAo+D than in C mice (*P <* 0.0001), D (*P <* 0.0001) or in pMCAo mice (*P <* 0.001). They were higher in pMCAo mice than in C or D mice (*P <* 0.0001). **f** The brain QUIN/KYNA ratio was higher in pMCAo+D mice than in all other groups (*P <* 0.0001). The ratio was significantly higher in pMCAo mice than in D (*P <* 0.0001) or C mice (*P <* 0.01)

We next assessed the KYN metabolites, KYNA and QUIN. Serum KYNA concentrations were significantly lower in pMCAo+D mice (104.4 ± 19.9 nM) than in D (235.0 ± 25.8 nM) or C mice (163.8 ± 15.2 nM) (*P <* 0.0001). They were also lower in pMCAo mice (124.7 ± 11.4 nM) than in D or C mice (*P <* 0.0001) as well as in C compared to D mice (*P <* 0.0001) (*n* = 9–10) (diabetes effect, *P =* 0.0001; stroke effect, *P <* 0.0001; diabetes*stroke, *P <* 0.0001) (Fig. 7a). Brain KYNA concentrations were lower in pMCAo+D mice than in D (*P <* 0.0001) or in C mice (*P <* 0.05). Brain KYNA levels were lower in pMCAo mice than in D (*P <* 0.0001) and lower in C mice than in D mice (*P <* 0.0001) (*n* = 7–8) (diabetes effect, *P <* 0.0001; stroke effect, *P <* 0.0001; diabetes*stroke, *P <* 0.0001) (Fig. 7d). Serum QUIN concentrations were significantly higher in pMCAo+D (452.0 ± 100.4 nM) and pMCAo mice (361.1 ± 65.7 nM) than in C (232.9 ± 10.8 nM) (*P <* 0.0001) or D mice (189.7 ± 17.4 nM) (*P <* 0.0001). QUIN levels were also higher in pMCAo+D mice than in pMCAo mice (*P <* 0.05) (*n* = 9–10) (diabetes effect, *P =* 0.032; stroke effect, *P <* 0.0001; diabetes*stroke, *P =* 0.032) (Fig. 7b). Brain QUIN levels were significantly higher in pMCAo+D than in C mice (*P <* 0.0001), D (*P <* 0.0001) or in pMCAo mice (*P <* 0.001). They were higher in pMCAo mice than in C (*P <* 0.0001) or D mice (*P <* 0.001) (*n* = 7–8) (diabetes effect, *P <* 0.0001; stroke effect, *P <* 0.0001; diabetes*stroke, *P <* 0.0001) (Fig. 7e). The serum QUIN/KYNA ratio was significantly higher in pMCAo+D mice (4.4 ± 1.0) than in pMCAo animals (2.9 ± 0.5), D (0.83 ± 0.12) or C mice (1.43 ± 0.10; *P <* 0.0001). The QUIN/KYNA ratio was higher in pMCAo than in C or D mice (*P <* 0.0001). There were no significant differences between C or D mice (*n* = 9–10) (diabetes effect, *P =* 0.012; stroke effect, *P <* 0.0001; diabetes*stroke, *P <* 0.0001) (Fig. 7c). The brain QUIN/KYNA ratio was higher in pMCAo+D mice than in all other groups (*P <* 0.0001). The ratio was significantly higher in pMCAo mice than in D (*P <* 0.0001) or C mice (*P <* 0.01) (*n* = 7–8) (diabetes effect, *P <* 0.01; stroke effect, *P <* 0.0001; diabetes*stroke, *P <* 0.0001) (Fig. 7f).

Overall, these data reveal significant increases in serum QUIN concentrations and QUIN/KYNA ratio in pMCAo+D mice compared to all other animal groups that mirror the same increased concentrations in the brain.

### pMCAo exacerbates neuronal death in ipsilateral hippocampus

**Fig. 8:**
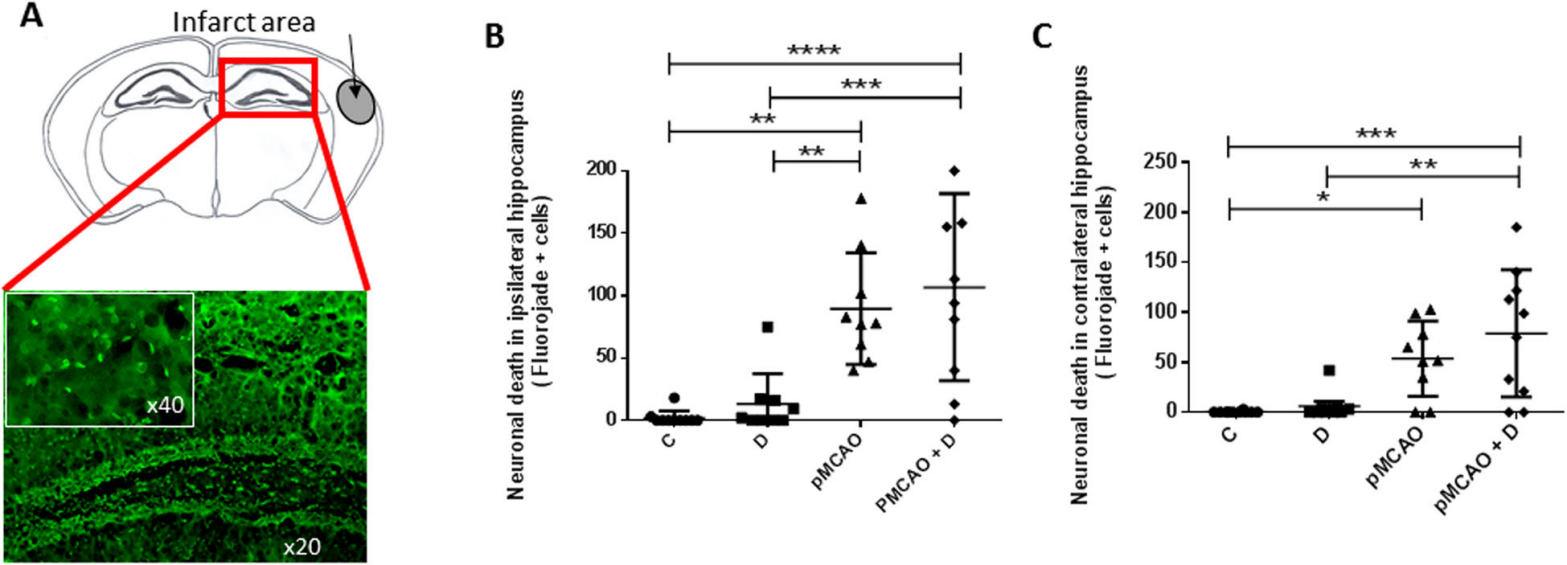
Neuronal degeneration in the ipsi- and contralateral hippocampus in C, D, pMCAo and pMCAo+D mice. Fluorojade staining was used to label degenerating cells in both hippocampus at D40. **a** Fluorojade staining of ipsilateral hippocampus in pMCAo+D mice (× 20 and magnification × 40). The diagram above shows the distant and restricted site of the induced infarct. **b** Numbers of Fluorojade+ neurons in ipsilateral hippocampus were significantly greater in pMCAo+D mice than in C (*P <* 0.0001) or D mice (*P <* 0.001). Neuronal degeneration was higher in pMCAo mice than in C or D mice (*P <* 0.01). **c** In contralateral hippocampus, numbers of degenerating neurons were higher in pMCAo+D mice than in C (*P <* 0.001) or D mice (*P <* 0.01). Numbers of Fluorojade+ neurons were significantly higher in pMCAo mice than in C mice (*P <* 0.05). *P < 0.05, **P < 0.01, ***P < 0.001, ****P < 0.0001

Fluorojade staining was used to label degenerating neurons at D40 after stroke (Fig. 8a). In ipsilateral hippocampus, numbers of degenerating cells were significantly higher in pMCAo+D mice (106.8 ± 75.0) than in C (2.1 ± 5.7) (*P <* 0.0001) or D mice (13.2 ± 24.2) (*P <* 0.001). There were significantly more degenerating neurons in pMCAo animals than in C or D mice (*P <* 0.01) (*n* = 9–10). There was no significant difference between pMCAo mice and pMCAo+D mice (89.6 ± 44.6) (*n* = 9–10) (Fig. 8b). This data suggests that neuronal degeneration in ipsilateral hippocampus is most strongly linked to the stroke effect (diabetes effect, *P =* 0.35; stroke effect, *P <* 0.0001; diabetes*stroke, *P =* 0.84).

A similar picture was evidenced in contralateral hippocampus. There were significantly more Fluorojade+ neurons in pMCAo+D mice (78.8 ± 63.5) than in C (0.3 ± 1.0) (*P <* 0.001) or D mice (6.1 ± 13.6) (*P <* 0.01). Numbers of Fluorojade+ cells were significantly higher in pMCAo (53.7 ± 37.6) than in C mice (*P <* 0.05) (*n* = 9–10) but differences between D and pMCAo mice were not significant (Fig. 8c) (diabetes effect, *P =* 0.22; stroke effect, *P <* 0.0001; diabetes*stroke, *P =* 0.44). Thus, stroke induction induced neuronal degeneration in both hippocampi, far from the infarct and peri-infarct area.

### Microglia/macrophage density is increased ipsilaterally in all pMCAo mice

**Fig. 9:**
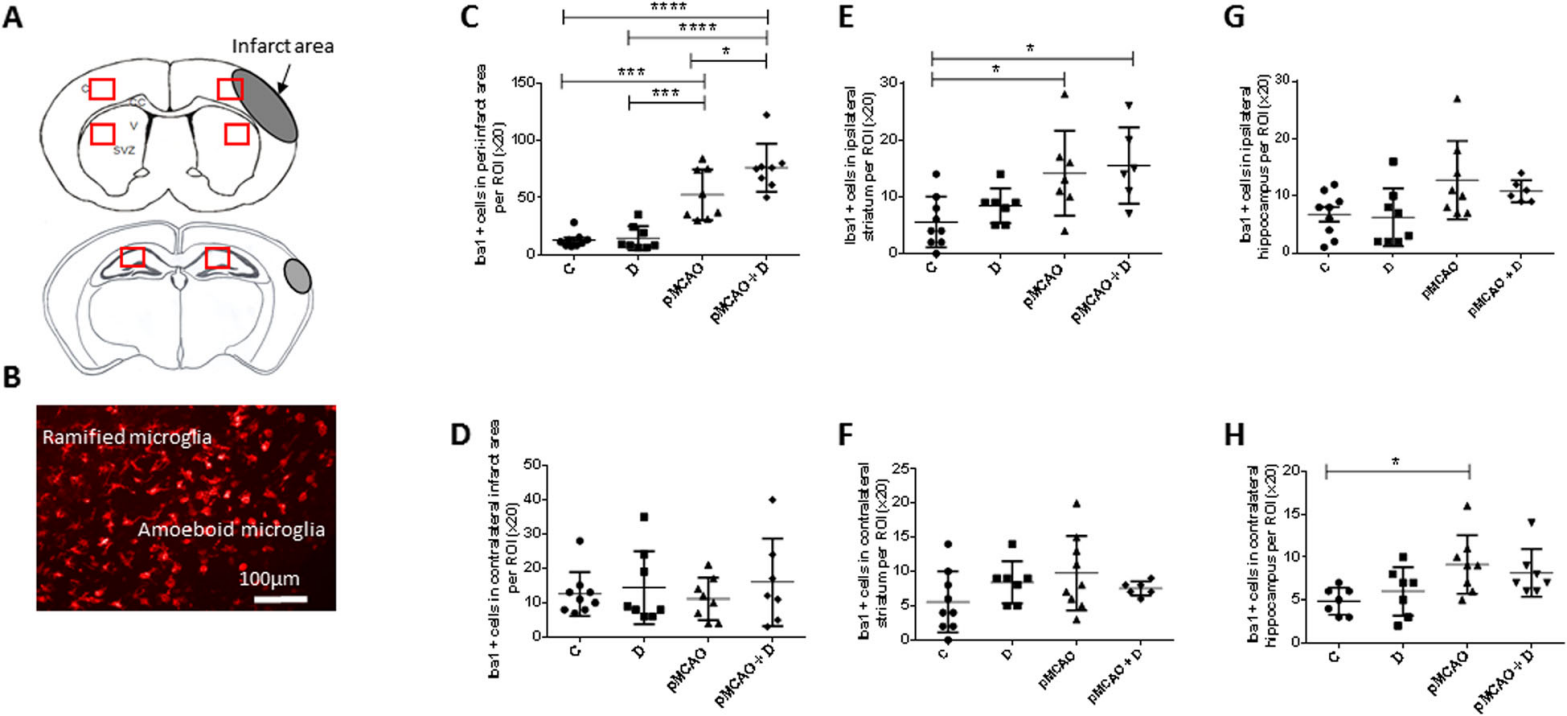
Microglial/macrophage infiltration is most strong in the ipsilateral peri-infarct area of pMCAo+D mice. Microglial/macrophages were stained by Iba1 immunostaining. **a** Schematic representation of the infarct area at 2 levels of a coronal brain section and regions (red boxes) from which numbers of stained cells were estimated. **c**, cortex; CC, corpus callosum; V, ventricle; SVZ, subventricular zone. **b** Iba1 immunostaining in pMCAo + D mice, ramified Iba1+ cells in the peri-infarct area and amoeboid Iba1+ cells in the core of the infarct; scale bar: 100 μm. **c** In the peri-infarct area, microglia/macrophages density was significantly higher in pMCAo+D mice than in C, D (*P <* 0.0001) or pMCAo mice (*P <* 0.05). Cell density was higher in pMCAo mice than in C or D mice (*P <* 0.001). **d** No significant differences were evidenced in the contralateral mirror region of the infarct. **e** Iba1+ cell density was higher in ipsilateral striatum of pMCAo+D mice than for C mice and in pMCAo mice than for C mice (*P <* 0.05). **f** No significant differences in cell density were detected in contralateral striatum for the different animal groups.**g** No significant differences in cell density were evidenced in ipsilateral hippocampus. **h** Iba1+ cell density was higher in contralateral hippocampus of pMCAo mice than in C mice. *P <* 0.05, *P < 0.05, ***P < 0.001, ****P < 0.0001

Microglia/macrophages were quantified as Iba1+ cells in the peri-infarct area, striatum and hippocampus, both ipsilateral and contralateral to the lesion (Fig. 9a, b). We hypothesize that inflammation may be mediated at sites remote from a unilateral cortical lesion by activated immune cells, or via cytokines they release.

In the peri-infarct area, microglia/macrophage density was significantly increased in pMCAo+D mice (76.0 ± 21.1 a.u.) compared to C (12.6 ± 6.4 a.u.), D (14.4 ± 10.6 a.u.) (*P <* 0.0001) or pMCAo mice (52.4 ± 22.3 a.u.) (*P <* 0.05). Density was higher in pMCAo mice than in C or D animals (*P <* 0.001) (*n* = 8–9) (diabetes effect, *P =* 0.0133; stroke effect, *P <* 0.0001; diabetes*stroke, *P =* 0.06) (Fig. 9c). We found no differences between groups in contralateral equivalent region (*n* = 7–9) (diabetes effect, *P =* 0.31; stroke effect, *P =* 0.98; diabetes*stroke, *P =* 0.64) (Fig. 9d).

In ipsilateral striatum, microglia/macrophage density was significantly increased in pMCAo+D (15.5 ± 6.7 a.u.) and pMCAo mice (14.1 ± 7.5 a.u.) compared to C mice (5.6 ± 4.4 a.u.) (*P <* 0.05). Anova analysis revealed stroke was most significantly linked to this observation (diabetes effect, *P =* 0.32; stroke effect, *P =* 0.0009; diabetes*stroke, *P =* 0.72) (*n* = 6–9) (Fig. 9e). We found no differences between groups in the contralateral striatum (*n* = 7–8) (diabetes effect, *P =* 0.87; stroke effect, *P =* 0.26; diabetes*stroke, *P =* 0.09) (Fig. 9f).

In ipsilateral hippocampus, microglia/macrophage density was not significantly different between animal groups (*n* = 6–9) (diabetes effect, *P =* 0.49; stroke effect, *P =* 0.0062; diabetes*stroke, *P =* 0.70) (Fig. 9g). In contralateral hippocampus, microglia/macrophage density was significantly increased in pMCAo mice (9.1 ± 3.4 a.u.) compared to C mice (4.9 ± 1.6 a.u.) (*P <* 0.05) (*n* = 7–8) (diabetes effect, *P =* 0.94; stroke effect, *P =* 0.0047; diabetes*stroke, *P =* 0.31) (Fig. 9h).

These data show that at D40, microglia/macrophage densities remain elevated in the peri-infarct area, less in remote regions such as striatum and not in hippocampus. Immune cell density was unaffected in contralateral brain regions, except in the hippocampus of stroke mice.

### pMCAo induces rarefaction of white matter in both striatum

**Fig. 10:**
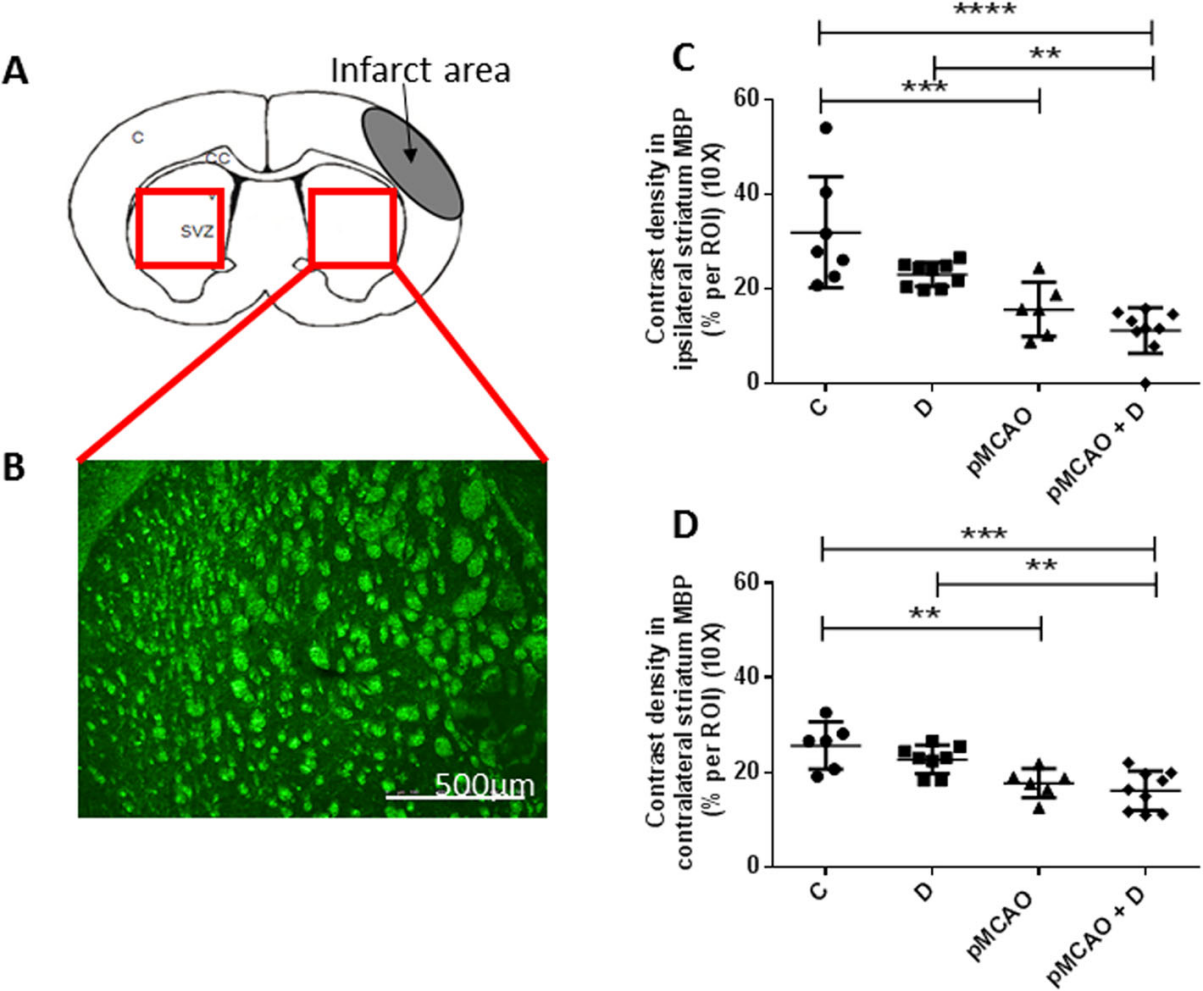
White matter density, assessed by myelin basic protein (MBP) immunostaining, decreases in ipsi- and contralateral striatum of pMCAo mice, diabetic or not. **a** Schematic representation of a coronal brain section showing the infarct region, the striatum and regions (red boxes) from which white matter density was estimated. **b** MBP immunostaining in the ipsilateral striatum of a pMCAo+D mouse, scale bar: 500 μm. **c** In the ipsilateral striatum, MBP+ cell density was significantly lower in pMCAo+D mice than in C (*P <* 0.0001) or D mice (*P <* 0.01) and lower in pMCAo mice than in C mice (*P <* 0.001). **d** MBP+ cell density in contralateral striatum was significantly lower in pMCAo+D mice than C (*P <* 0.001) or D mice (*P <* 0.01) and lower in pMCAo mice than in C mice (*P <* 0.01). **P < 0.01, ***P < 0.001, ****P < 0.0001

We quantified MBP+ cells in both striatum to provide an index of white matter density (Fig. 10a, b). MBP+ cells contrast density was significantly lower in the ipsilateral striatum of pMCAo+D mice (11.2 ± 4.9 a.u.) than in C (31.9 ± 11.7 a.u.) (*P <* 0.0001) or D mice (23.0 ± 2.5 a.u.) (*P <* 0.01). MBP+ cell contrast density was lower in pMCAo (15.6 ± 5.7 a.u.) than in C mice (*P <* 0.001) (*n* = 6–9) (diabetes effect, *P =* 0.01; stroke effect, *P <* 0.0001; diabetes*stroke, *P =* 0.36) (Fig. 10c).

In contralateral striatum, MBP+ cells contrast density was significantly lower in pMCAo+D (16.1 ± 4.2 a.u.) than in C (25.7 ± 5.0 a.u.) (*P <* 0.001) or D mice (22.8 ± 3.0 a.u.) (*P <* 0.01). MBP+ cell contrast density was lower in pMCAo (17.7 ± 3.1 a.u.) than in C mice (*P <* 0.01) (*n* = 6–9) (diabetes effect, *P =* 0.14; stroke effect, *P <* 0.0001; diabetes*stroke, *P =* 0.64) (Fig. 10d).

### Serum metabolites of the kynurenine pathway correlate with functional and structural changes in PSCI mice

Serum IDO activity was negatively correlated (r = − 0.4788, *P =* 0.0056) with the anxiety index from the open field test, whereas serum QUIN (r = 0.3984, *P =* 0.0239) and serum QUIN/KYNA (r = 0.4218, *P =* 0.0162) were positively correlated and serum KYNA was negatively correlated (r = − 0.4398, *P =* 0.0118) with spatial learning during the retention phase of the Barnes maze test (Table 3). None of the serum levels was correlated with indices from the reversal phase. No correlations were made with brain kynurenine metabolites or with LTP data since experiments were made on separate sets of mice.

**Table 3:**
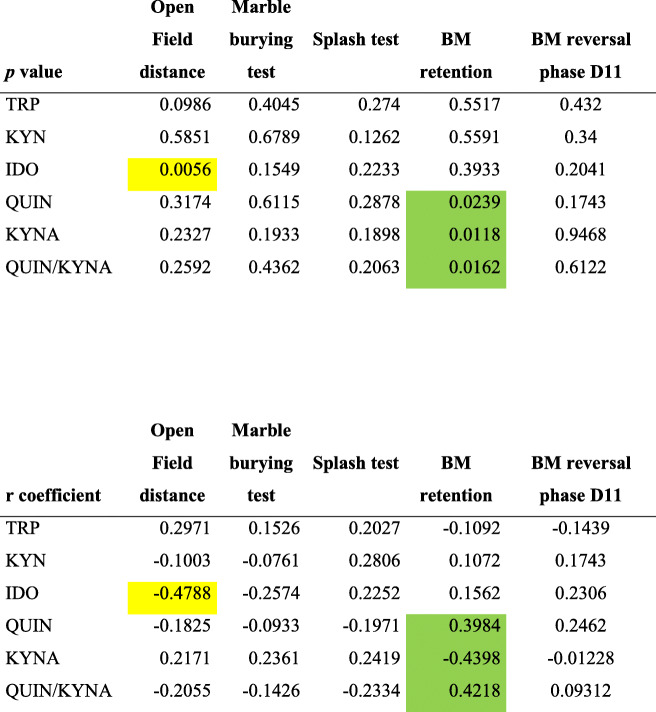
Correlations between TRP (Tryptophan), KYN (Kynurenine), IDO (indoleamine 2,3 dioxygenase) activity, QUIN (Quinolinic acid), KYNA (Kynurenic acid), QUIN/KYNA serum levels and distance mouse travelled in open field, marble burying test, splash test, retention and reversal phases of the Barnes maze (*P <* 0.05 in green, *P <* 0.01 in yellow; not significant uncoloured). BM, Barnes maze

While there were no strong correlations between IDO activity and morphological changes, QUIN and QUIN/KYNA ratio were significantly correlated with neuronal death in the hippocampus, microglia/macrophage density in the peri-infarct area, ipsilateral striatum and both hippocampi, as well as white matter rarefaction in both striatums (Table 4). Inversely, KYNA was negatively correlated with neuronal death, microglia/macrophage density in the peri-infarct area, ipsilateral striatum and both hippocampi, and white matter rarefaction in both striatums (Table 4). Correlations within each group are found in supplementary Tables 1 to 8.

**Table 4:**
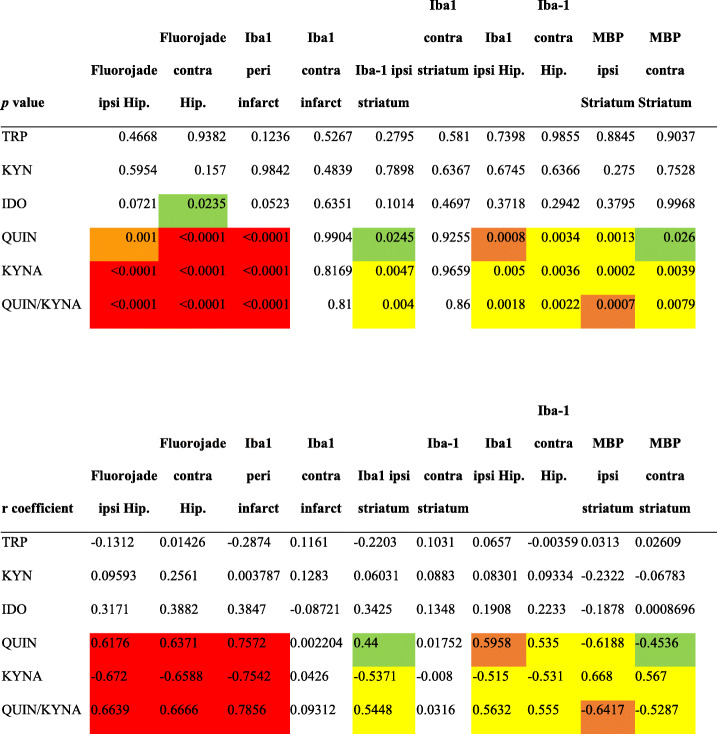
Correlations between TRP (Tryptophan), KYN (Kynurenine), IDO (indoleamine 2,3 dioxygenase) activity, QUIN (Quinolinic acid), KYNA (Kynurenic acid), QUIN/KYNA serum levels and neuronal death, microglia/macrophages infiltration, and white matter density. Ipsi, ipsilateral; Hip, hippocampus; Contra, contralateral; MBP, Myelin Basic Protein (*P <* 0.05 in green, *P <* 0.01 in yellow, *P <* 0.001 in orange, *P <* 0.0001 in red; not significant uncolored)

### Post-stroke patients with cognitive decline present higher levels of serum KYN, IDO activity, QUIN concentrations and QUIN/KYNA ratio

Clinical data and blood samples of twenty-three stroke patients at admission were analysed. Thirteen patients showed a post-stroke cognitive dysfunction. Demographic and clinical data were not significantly different between groups (Table 5).

**Table 5:** Demographic and clinical data of patients without (C-) or with (C+) post-stroke cognitive decline. F, female; M, male

	C- patients (n = 10)	C+ patients (*n* = 13)	*P*
Gender (F/M)	4/6	5/8	1
Mean age ± SD (years)	64.7 ± 13.3	69.4 ± 17.8	0.38
Diabetes	1	2	1
Hypertension	5	8	0.685
Fibrinolysis	8	12	0.5596
Thrombectomy	5	6	1
Anterior circulation	7	13	0.4561
Posterior circulation	3	0	0.0678
Cardio-embolic	4	6	1
Atheroma	4	5	1
Undetermined	2	2	1

**Fig. 11:**
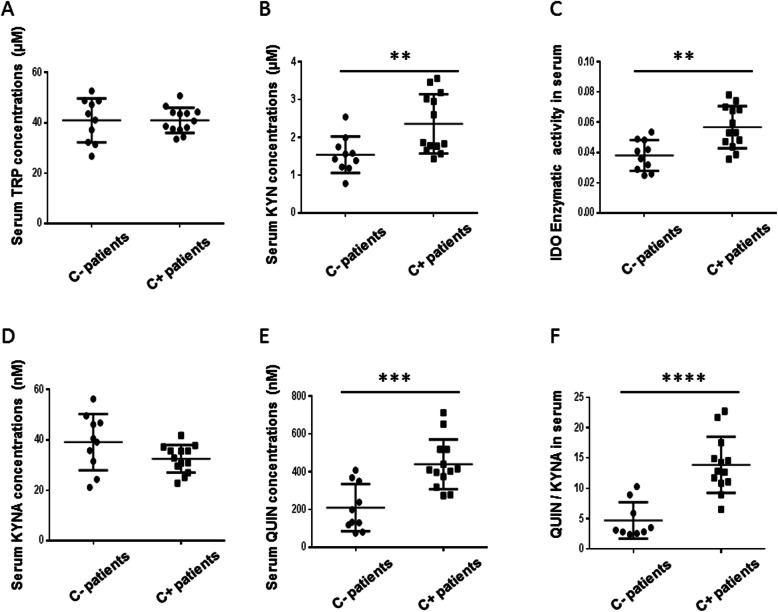
Serum changes of the tryptophane (TRP) (**a**) and kynurenine (KYN) (**b**) concentrations, indoleamine 2, 3-dioxygenase (IDO) activity (**c**), kynurenic acid (KYNA) (**d**) and quinolinic acid (QUIN) (**e**) concentrations and QUIN/KYNA ratios (**f**) in patients without (C-) or with (C+) cognitive decline. There were no significant differences in serum TRP (**a**) or KYNA concentrations (**d**) between patients. Serum levels of KYN (**b**) (*P* < 0.01), IDO enzymatic activity (**c**) (*P* < 0.01), QUIN (**e**) (*P* < 0.001), and QUIN/KYNA (**f**) (*P* < 0.0001) were significantly elevated in the C+ group compared to the C- group. **P < 0.01, ***P < 0.001, ****P < 0.0001

There were no significant differences in serum TRP levels in patients with cognitive dysfunction (C+) (41.0 ± 5.0 μM) compared to patients without cognitive dysfunction (C^−^) (40.9 ± 8.7 μM) (effect size 0.0025). Serum KYN levels were significantly elevated in the C+ group (2.4 ± 0.8 μM) versus the C- group (1.5 ± 0.5 μM) (*P* = 0.004) (effect size 0.46), as was serum activity of IDO (0.057 ± 0.014 versus 0.038 ± 0.010, respectively) (*P* = 0.0019) (effect size 0.40). Serum KYNA concentrations were not different between C+ (32.5 ± 5.5 nM) and C- (39.1 ± 11.2 nM) groups (effect size 0.19). Serum QUIN concentrations were significantly higher in the C+ (439.4 ± 131.9 nM) than in the C- group (210.4 ± 114.9 nM) (*P* = 0.0004) (effect size 0.67). The serum QUIN/KYNA ratio was significantly higher in the C+ (13.9 ± 4.6) than in the C- group (4.7 ± 3) (*P* < 0.0001) (effect size 0.88) (Fig. 11).

The results of this retrospective unselected cohort suggest that initial serum KYN and QUIN levels, IDO activity and QUIN/KYNA ratio, at the acute phase of stroke, are significantly different between the patients who will or will not present a cognitive dysfunction.

## Discussion

This study has shown that as PSCI developed in diabetic mice within 6 weeks after a cortical infarct, LTP in ipsilateral and contralateral hippocampus was abolished. This model of PSCI after stroke coupled with diabetes, a major vascular risk factor, showed several morphological features: **(i**) increased neuronal death in both hippocampi, (**ii**) increased microglia/macrophage density in peri-infarct area and (**iii**) white matter rarefaction in both striatum. Furthermore, serum QUIN levels and QUIN/KYNA ratios were increased and found positively correlated with degraded cognitive function on a spatial memory task. Moreover, these same serum biomarkers in a small unselected cohort of stroke patients were found to predict cognitive dysfunction in those patients. These molecules may lead to new serum biomarkers of cognitive dysfunction in stroke patients.

As recently determined in the Oxford Vascular Study, the 5-year risk of dementia after stroke was associated with only one vascular risk factor, diabetes [9], a condition whose prevalence is projected to double by 2030 [38]. Factors underlying this association are not clear. Diabetic microangiopathy and impaired vasoreactivity are correlated to stroke incidence and dementia and might contribute to inappropriate neurovascular coupling [38–40]. Middle-aged rats subjected to nicotinamide–streptozotocin injection and embolic middle cerebral artery occlusion developed a cognitive deficit 2 months after diabetes induction and one month after stroke, reduced spine density and dendritic arborisation in hippocampal neurons and aggravated neurovascular disruption [18]. Moreover, we have previously shown an unbalance between brain pro- and anti-inflammatory cytokines in diabetic mice subjected to pMCAo, with a shift towards a higher inflammatory response (IL-1β, MCP1, IL-6 and TNFα) [21]. This pro-inflammatory shift could explain aggravating effects of diabetes in post-stroke recovery of patients [41] and rodents [16]. It may be linked to the increased IDO activity detected in diabetic mice. IDO, a key enzyme in the conversion of TRP into KYN is activated by pro-inflammatory cytokines such as IFNγ, IL-1, IL-6 [42], and inhibited by anti-inflammatory cytokines such as IL-4 [43]. IDO activity is then assumed to reflect inflammation [44]. Our data support this view in that IDO activity levels were correlated with the open field index of anxiety, which was enhanced in diabetic mice with or without stroke. IDO activity was not correlated with spatial memory impairment, neuronal loss, microglia/macrophage proliferation or white matter rarefaction. This finding contrasts with a clinical study [22] suggesting that IDO activity is correlated to cognitive decline at one month post-stroke. Direct comparison is difficult since while 27% of studied patients were diabetic, 85% of patients were hypertensive and little is known on how distinct vascular risk factors affect IDO activity.

As serum IDO did not discriminate between mice with or without post-stroke cognitive impairment, we next studied metabolites produced by the kynurenine pathway, including QUIN and KYNA active in glutamatergic transmission. QUIN, largely produced by activated microglia/macrophages, is a neurotoxic NMDA receptor agonist [23, 24]. KYNA, mainly produced by astrocytes [45], in contrast is an NMDA receptor antagonist. It is considered neuro-protective [25], as it attenuates ischemia induced learning deficits in the rat [46]. We found serum and brain QUIN levels were highest in stroke-diabetic mice. In these animals, numbers of microglia/macrophages, which produce QUIN, were significantly higher in the peri-infarct area [47]. Conversely, serum KYNA levels were significantly decreased in pMCAo mice with or without diabetes. We thus evaluated the ratio of these two KYN metabolites, QUIN/KYNA, which was dramatically higher in stroke-diabetic mice than in all other groups. The serum ratio was positively correlated with degraded spatial memory performance, hippocampal neuronal death and microglia/macrophage infiltration in both hippocampi, the peri-infarct and ipsilateral striatal areas, and white matter rarefaction. Serum QUIN levels were also positively correlated with these indices, while correlations were negative for serum KYNA concentrations.

Interestingly, these serum kynurenine pathway metabolites closely mirrored brain concentrations. They may contribute to our data on synaptic plasticity, even though our data do not permit proper comparison since LTP was tested in a separate set of mice. An enhanced QUIN/KYNA ratio could favor neurotoxic rather than protective effects with an increase in NMDA receptor activation. Recent work with the pMCAo mice model, in the absence of diabetes, revealed remodelling of hippocampal circuitry consequent on stroke-induced neurogenesis. These data supported the loss of hippocampus-dependent memory, but synaptic plasticity was not examined [48].

We evaluated hippocampal-dependent spatial memory [49] using the Barnes maze test as in our previous works on models of Alzheimer’s disease and stroke [21, 50]. Strikingly, we found impaired performances in the retention and reversal phases of the task were associated with suppression of LTP in ipsi- and contralateral hippocampus in pMCAo mice, with or without diabetes. These data imply that cerebral ischemia exerts sustained actions over time, at a distance from the infarct area. Possibly products of biochemical cascades triggered by ischemia diffused to both hippocampi, induced neuronal death and suppressed synaptic plasticity. We suggest that inflammatory signalling induced by stroke and enhanced by diabetes [21], evidenced here as higher serum QUIN levels and QUIN/KYNA ratios, should be a target of novel stroke therapies. For instance, peripheral administration of the soluble TNF inhibitor XPro1595 has been shown to rescue impaired LTP in 5xFAD mice, a mouse model of Alzheimer’s disease, together with a decreased beta-amyloid load [51]. Further, we have shown that long term memory impairment is prevented by the immunomodulatory drug Glatiramer acetate [21], already FDA approved in multiple sclerosis. Overall, these data confirm a key role for inflammation in cognitive decline. QUIN inhibitors administration may be an alternative approach and we plan to study this question in detail.

Ischemic stroke was modelled in this work using the pMCAo model rather than using the ischemia/reperfusion model transient MCAo (tMCAO). This choice ensured that the infarct spared the hippocampus. Moreover, mortality is much lower in diabetic pMCAo mice than in diabetic tMCAo animals. A further advantage of the pMCAo model is that mice had completely recovered from sensorimotor deficit when cognitive assessment was made, so data of the Barnes maze test could be interpreted without bias. We wished also to develop a clinically relevant model and post-stroke recanalization induced in the tMCAO procedure is present in only a minority of patients [52]. Moreover, even small ischemic cortical lesions are associated with significant cognitive impairment in animal models [53, 54] as in patients [9, 55] as we have shown here.

While the mechanisms of PSCI are not completely resolved, our data have shown a strong link to the kynurenine pathway. Within this pathway, we showed serum and brain levels of TRP did not differ between groups. Serotonin signalling has been linked to post-stroke recovery [56], since selective serotonin reuptake inhibitors improve motor function in stroke patients [57], and enhance excitatory synaptic transmission in rat hippocampus [32]. However, such serotonin based treatments are still debated. Discrepancies in trials of these therapies have been attributed to heterogeneity between protocols, methodological limitations [56] and the small cohort sizes [58]. Our data also suggest that the TRP downstream metabolites should rather be targeted rather than serotonin and that this could explain the lack of efficiency in some trials. In the clinical study we conducted next, we showed that this pathway is promising, but needs larger multicentre confirmatory studies. Indeed, in this small cohort, diabetes was only found in 3 out of the 23 patients and its role per se in the development of the cognitive impairement remains difficult to assess as we did in our model. Another hypothesis would be that diabetes in our murine model increases the inflammatory post-stroke status, that might exist in humans, whatever the vascular risk factor. Our working hypothesis was that post-stroke inflammation may trigger remote and delayed dysfunctions and lesions responsible for cognitive decline as also suggested by the work performed by Doyle et al. [53]. It could be that in our mouse stroke model, diabetes ensures a higher level of post-stroke brain inflammation as we have shown previously [21] and that the level of inflammation is comparable to the one encountered in older patients who may also combine other comorbidities (hypertension, metabolic syndrome, etc) that could also contribute to increased inflammation. This hypothesis is reinforced by culture cells experiments performed by Du et al. [59], showing an interspecies difference in the inflammatory response of microglial cells in conditions of oxygen-glucose deprivation, human cells upregulating a higher number of genes involved in inflammation than mouse cells. The additive impact of age, comorbidities and vascular risk factors in particular on brain inflammation and subsequently PSCI has yet to be determined. Moreover, as all patients were admitted for fibrinolysis and potential thrombectomy because of proximal artery occlusion indicates that this selected cohort is more severe than the classical stroke population. At last, as clinical data were collected retrospectively, the neuropsychological investigation was very heterogeneous; we cannot exclude either that some of the patients already presented a cognitive decline before stroke. A new larger study to confirm these data should be designed prospectively.

Clinically, the kynurenine pathway has been linked to Alzheimer’s disease. KYNA levels in CSF have been shown to be significantly reduced, although QUIN concentrations were not elevated [60]. Further, QUIN immunoreactivity has been shown to be enhanced in the hippocampus of patients with Alzheimer’s disease [61]. In this study, changes in the QUIN level and the QUIN/KYNA ratio were robust and we suggest they may serve as a reliable biomarker for PSCI in patients. They fulfil all the necessary criteria for a biomarker: they are released from damaged tissue and elevated in blood, reproducible fashion providing an index, which can be easily measured and interpreted and serve as reliable indicator of pathogenic processes [62].

We should note some limitations of the present study. While our current data show that IDO activity increases in stroke-diabetic mice and previous work has revealed enhanced levels of pro-inflammatory cytokines in stroke-diabetic mice [21], further details on innate and adaptive immune responses in the hippocampus in particular, are needed to completely characterize PSCI inflammation. We did not either directly evaluate the essential pyridine nucleotide end product, NAD+, produced by the kynurenine pathway and with a major role in cell homeostasis [63]. At last, the discrepancy of sex and age between mice (only young adult males) in our preclinical study and the cohort of patients in early 60s, including females and males, may limit the translational impact of our study. Our primary aim was to build a PSCI preclinical model that shares common characteristics with those encountered in patients and then, in a translational purpose, we wished to validate experimental data on QUIN and QUIN/KYNA in a small exploratory cohort of patients. Despite sex and age differences between the two species, we found similar modifications of the biomarkers QUIN and QUIN/KYNA in mice and in patients with PSCI. Nevertheless, a large prospective clinical study is mandatory to confirm these preliminary results and the effects of age and sex should be specifically studied in mice, as recommended by the STAIR preclinical recommendations [64].

## Conclusions

This well characterized PSCI model is a close mimic of the clinical setting and accurately encompasses pathophysiological aspects of the syndrome. It will provide a realistic setting to develop a specific QUIN inhibitor as proposed in one preclinical work [46]. There are currently no therapeutic options to cure or prevent PSCI. Limiting the size of the triggering stroke is not sufficient [9]. Large prospective clinical studies are now needed to confirm our preliminary data in stroke patients, which show that serum QUIN concentrations and QUIN/KYNA ratios are reliable biomarkers to predict PSCI and ask whether these indices are predictive biomarkers of a higher risk or severity of the syndrome. Clinical work to provide a useful biomarker will most importantly need to define an accurate cut-off value beyond which the serum biomarker is reliably correlated with PSCI development.

## Supplementary Information

**Additional file 1.**

## Abbreviations

aCSF: Artificial cerebrospinal fluid
BM: Barnes maze
C: Control mice
CA1: Cornu ammonis 1
D: Diabetes
fEPSP: Field excitatory post synaptic potential
Iba1: Ionized calcium binding adaptor molecule 1
IDO: Indolamine 2,3-dioxygénase
IFN: InTERFERON
IL: Interleukin
IP: Intra-peritoneal
KYN: Kynurenine
KYNA: Kynurenic acid
LTP: Long-term potentiation
MBP: Myelin basic protein
MEA: Multi-electrode array
MRI: Magnetic resonance imaging
NMDA: N-methyl-D-aspartic acid
pMCAo: Permanent middle cerebral artery occlusion
pMCAo+D: Permanent middle cerebral artery occlusion in diabetic mice
PSCI: Post stroke cognitive impairment
QUIN: Quinolinic acid
TRP: Tryptophan
VCI: Vascular cognitive impairment
