# Supplementary material for: Increased serum QUIN/KYNA is a reliable biomarker of post-stroke cognitive decline
Source: Mol Neurodegener. 2021 Feb 15;16:7. doi: 10.1186/s13024-020-00421-4 (PMC7885563; doi:10.1186/s13024-020-00421-4)
Supplement: Supplementary file 1 — Additional file 1. [file 13024_2020_421_MOESM1_ESM.docx]

**Supplementary Tables**

**Supplementary Table 1.** Correlations within the group Control (C) between TRP (Tryptophan), KYN (Kynurenine), IDO (indoleamine 2,3 dioxygenase) activity, QUIN (Quinolinic acid), KYNA (Kynurenic acid), QUIN/KYNA serum levels and distance mouse travelled in open field, marble burying test, splash test, retention and reversal phases of the Barnes maze (*P <* 0.05 in green; not significant uncoloured). BM, Barnes maze.

| ***p* value** | | **Open Field distance** | **Marble**  **burying**  **test** | | **Splash test** | | **BM retention** | | | **BM reversal phase D11** | | |  |  |  |
| --- | --- | --- | --- | --- | --- | --- | --- | --- | --- | --- | --- | --- | --- | --- | --- |
| TRP | | 0.7435 | 0.6188 | | 0.4408 | | 0.7520 | | | 0.3363 | | |  |  |  |
| KYN | | 0.1080 | 0.8952 | | 0.8525 | | 0.2992 | | | 0.1206 | | |  |  |  |
| IDO | | 0.0108 | 0.9942 | | 0.3264 | | 0.0468 | | | 0.2499 | | |  |  |  |
| QUIN | | 0.8536 | 0.3878 | | 0.8993 | | 0.5088 | | | 0.8005 | | |  |  |  |
| KYNA | | 0.0776 | 0.7488 | | 0.1559 | | 0.4704 | | | 0.6197 | | |  |  |  |
| QUIN/KYNA | | 0.0589 | 0.2711 | | 0.1409 | | 0.4618 | | | 0.3363 | | |  |  |  |
|  | |  |  | |  | |  | |  | | |  | | |  |
|  | |  |  | |  | |  | |  | | |  | | |  |
| **r coefficient** | | **Open**  **Field distance** | **Marble**  **burying**  **test** | | **Splash test** | | **BM retention** | | | **BM reversal phase D11** | | |  |  |  |
| TRP | | 0.1333 | 0.1924 | | -0.2929 | | 0.1429 | | | -0.3667 | | |  |  |  |
| KYN | | 0.5833 | 0.05246 | | -0.07531 | | -0.4246 | | | -0.5667 | | |  |  |  |
| IDO | | 0.8167 | 0.008744 | | 0.3887 | | -0.7381 | | | -0.4333 | | |  |  |  |
| QUIN | | -0.07531 | 0.3249 | | 0.05042 | | 0.2857 | | | 0.1004 | | |  |  |  |
| KYNA | | -0.1229 | -0.1229 | | 0.4958 | | 0.2994 | | | 0.11925 | | |  |  |  |
| QUIN/KYNA | | 0.6667 | 0.4807 | | -0.5356 | | -0.3095 | | | -0.3667 | | |  |  |  |
|  | |  |  | |  | |  | |  | | |  | | |  |
|  |  | |  |  | |  | |  | | |  | | |  | |

**Supplementary Table 2.** Correlations within the group Control (C) between TRP (Tryptophan), KYN (Kynurenine), IDO (indoleamine 2,3 dioxygenase) activity, QUIN (Quinolinic acid), KYNA (Kynurenic acid), QUIN/KYNA serum levels and neuronal death, microglia/macrophages infiltration, and white matter density. Ipsi, ipsilateral; Hip, hippocampus; Contra, contralateral; MBP, Myelin Basic Protein (*P <* 0.05 in green; not significant uncolored).

| ***p* value** | **Fluorojade ipsi Hip.** | **Fluorojade contra Hip.** | **Iba1**  **peri infarct** | **Iba1**  **contra infarct** | **Iba-1 ipsi striatum** | **Iba1**  **contra striatum** | **Iba1**  **ipsi Hip.** | **Iba-1**  **contra Hip.** | **MBP**  **ipsi Striatum** | **MBP**  **contra Striatum** |  |  |  |  |
| --- | --- | --- | --- | --- | --- | --- | --- | --- | --- | --- | --- | --- | --- | --- |
| TRP | 0.4167 | 0.9999 | 0.8691 | 0.4121 | 0.4559 | 0.4666 | 0.0260 | 0.3952 | 0.9194 | 0.75 |  |  |  |  |
| KYN | 0.9999 | 0.9999 | 0.3995 | 0.2414 | 0.3258 | 0.3038 | 0.2249 | 0.6063 | 0.3556 | 0.9167 |  |  |  |  |
| IDO | 0.3611 | 0 | 0.0659 | 0.8888 | 0.6495 | 0.9746 | 0.2051 | 0.9794 | 0.0167 | 0.4167 |  |  |  |  |
| QUIN | 0.3056 | 0 | 0.9966 | 0.2143 | 0.5877 | 0.2432 | 0.4975 | 0.9754 | 0.8028 | 0.9167 |  |  |  |  |
| KYNA | 0.6250 | 0 | 0.8999 | 0.8731 | 0.0825 | 0.9436 | 0.4790 | 0.9722 | 0.1556 | 0.75 |  |  |  |  |
| QUIN/KYNA | 0.9444 | 0 | 0.9030 | 0.8009 | 0.0713 | 0.9397 | 0.1273 | 0.7857 | 0.0583 | 0.75 |  |  |  |  |
|  |  |  |  |  |  |  |  |  |  |  |  |  |  |  |
|  |  |  |  |  |  |  |  |  |  |  |  |  |  |  |
| **r coefficient** | **Fluorojade ipsi Hip.** | **Fluorojade contra Hip.** | **Iba1**  **peri infarct** | **Iba1**  **contra infarct** | **Iba1 ipsi striatum** | **Iba-1**  **contra striatum** | **Iba1**  **ipsi Hip.** | **Iba-1**  **contra Hip.** | **MBP**  **ipsi striatum** | **MBP**  **contra striatum** |  |  |  |  |
| TRP | 0.3195 | 0 | 0.06723 | 0.3109 | -0.2857 | -0.2773 | 0.7448 | 0.3819 | 0.08571 | -0.4 |  |  |  |  |
| KYN | -0.62282 | 0 | 0.3193 | 0.437 | ­-0.3698 | -0.3866 | 0.4603 | 0.2364 | 0.4857 | -0.2 |  |  |  |  |
| IDO | -0.3651 | 0 | 0.6471 | 0.05883 | -0.1765 | 0.01689 | -0.4686 | 0.01818 | 0.9429 | 0.6 |  |  |  |  |
| QUIN | -0.3912 | 0 | 0.004237 | 0.4576 | -0.2076 | -0.4322 | -0.2874 | -0.01835 | 0.1429 | -0.2 |  |  |  |  |
| KYNA | -0.1948 | 0 | -0.0563 | -0.06329 | -0.616 | 0.02984 | 0.2689 | 0.01835 | -0.6667 | -0.4 |  |  |  |  |
| QUIN/KYNA | 0.04564 | 0 | -0.05642 | 0.1008 | 0.6387 | -0.03361 | -0.2176 | 0.1273 | 0.8286 | 0.4 |  |  |  |  |

**Supplementary Table 3.** Correlations within the diabetic group (D) between TRP (Tryptophan), KYN (Kynurenine), IDO (indoleamine 2,3 dioxygenase) activity, QUIN (Quinolinic acid), KYNA (Kynurenic acid), QUIN/KYNA serum levels and distance mouse travelled in open field, marble burying test, splash test, retention and reversal phases of the Barnes maze (*P <* 0.05 in green; not significant uncoloured). BM, Barnes maze.

| ***p* value** | | **Open Field distance** | **Marble**  **burying**  **test** | | **Splash test** | | **BM retention** | | | **BM reversal phase D11** | | |  |  |  |
| --- | --- | --- | --- | --- | --- | --- | --- | --- | --- | --- | --- | --- | --- | --- | --- |
| TRP | | 0.9999 | 0.5560 | | 0.9190 | | 0.4976 | | | 0.4048 | | |  |  |  |
| KYN | | 0.9635 | 0.1389 | | 0.9516 | | 0.9635 | | | 0.3778 | | |  |  |  |
| IDO | | 0.1667 | 0.2 | | 0.6397 | | 0.9635 | | | 0.6357 | | |  |  |  |
| QUIN | | 0.5825 | 0.8873 | | 0.6837 | | 0.4048 | | | 0.0153 | | |  |  |  |
| KYNA | | 0.3026 | 0.3956 | | 0.9190 | | 0.6615 | | | 0.0508 | | |  |  |  |
| QUIN/KYNA | | 0.6615 | 0.2357 | | 0.9516 | | 0.1429 | | | 0.8873 | | |  |  |  |
|  | |  |  | |  | |  | |  | | |  | | |  |
|  | |  |  | |  | |  | |  | | |  | | |  |
| **r coefficient** | | **Open**  **Field distance** | **Marble**  **burying**  **test** | | **Splash test** | | **BM retention** | | | **BM reversal phase D11** | | |  |  |  |
| TRP | | 0 | -0.2857 | | -0.05406 | | 0.3214 | | | 0.3784 | | |  |  |  |
| KYN | | -0.03871 | -0.6429 | | -0.03604 | | 0.03571 | | | 0.3964 | | |  |  |  |
| IDO | | 0.6071 | 0.5714 | | -0.2162 | | 0.03571 | | | 0.2162 | | |  |  |  |
| QUIN | | 0.2523 | 0.2523 | | -0.1818 | | -0.3784 | | | 0.8727 | | |  |  |  |
| KYNA | | 0.4643 | -0.3929 | | -0.05406 | | -0.2143 | | | 0.7748 | | |  |  |  |
| QUIN/KYNA | | -0.2143 | 0.5357 | | 0.03664 | | 0.7825 | | | -0.07207 | | |  |  |  |
|  | |  |  | |  | |  | |  | | |  | | |  |
|  |  | |  |  | |  | |  | | |  | | |  | |

**Supplementary Table 4.** Correlations within the diabetic group (D) between TRP (Tryptophan), KYN (Kynurenine), IDO (indoleamine 2,3 dioxygenase) activity, QUIN (Quinolinic acid), KYNA (Kynurenic acid), QUIN/KYNA serum levels and neuronal death, microglia/macrophages infiltration, and white matter density. Ipsi, ipsilateral; Hip, hippocampus; Contra, contralateral; MBP, Myelin Basic Protein (*P <* 0.05 in green; not significant uncolored).

| ***p* value** | **Fluorojade ipsi Hip.** | **Fluorojade contra Hip.** | **Iba1**  **peri infarct** | **Iba1**  **contra infarct** | **Iba-1 ipsi striatum** | **Iba1**  **contra striatum** | **Iba1**  **ipsi Hip.** | **Iba-1**  **contra Hip.** | **MBP**  **ipsi Striatum** | **MBP**  **contra Striatum** |  |  |  |  |
| --- | --- | --- | --- | --- | --- | --- | --- | --- | --- | --- | --- | --- | --- | --- |
| TRP | 0.2548 | 0.0619 | 0.7111 | 0.9417 | 0.9999 | 0.9619 | 0.6222 | 0.0778 | 0.2548 | 0.3024 |  |  |  |  |
| KYN | 0.8872 | 0.2845 | 0.7778 | 0.7353 | 0.6 | 0.8048 | 0.7111 | 0.2444 | 0.6615 | 0.3536 |  |  |  |  |
| IDO | 0.6997 | 0.4869 | 0.7778 | 0.0756 | 0.9999 | 0.7714 | 0.6222 | 0.3444 | 0.0881 | 0.6615 |  |  |  |  |
| QUIN | 0.6652 | 0.1988 | 0.3028 | 0.7050 | 0.9 | 0.7881 | 0.1444 | 0.8333 | 0.1437 | 0.1730 |  |  |  |  |
| KYNA | 0.5973 | 0.8143 | 0.6333 | 0.9198 | 0.9999 | 0.0190 | 0.0722 | 0.9222 | 0.4444 | 0.0238 |  |  |  |  |
| QUIN/KYNA | 0.3125 | 0.5833 | 0.1556 | 0.4438 | 0.6 | 0.4438 | 0.4111 | 0.7778 | 0.5948 | 0.3956 |  |  |  |  |
|  |  |  |  |  |  |  |  |  |  |  |  |  |  |  |
|  |  |  |  |  |  |  |  |  |  |  |  |  |  |  |
| **r coefficient** | **Fluorojade ipsi Hip.** | **Fluorojade contra Hip.** | **Iba1**  **peri infarct** | **Iba1**  **contra infarct** | **Iba1 ipsi striatum** | **Iba-1**  **contra striatum** | **Iba1**  **ipsi Hip.** | **Iba-1**  **contra Hip.** | **MBP**  **ipsi striatum** | **MBP**  **contra striatum** |  |  |  |  |
| TRP | 0.4636 | 0.7103 | -0.2029 | -0.03615 | 0.2236 | 0.03742 | 0.2648 | -0.7945 | 0.4636 | 0.4643 |  |  |  |  |
| KYN | 0.07319 | 0.4312 | -0.1449 | -0.4446 | -0.4472 | -0.1123 | 0.1765 | -0.5591 | 0.2143 | 0.4286 |  |  |  |  |
| IDO | -0.1708 | 0.2917 | 0.1449 | -0.6747 | -0.2236 | 0.1497 | 0.2648 | -0.4708 | -0.1708 | 0.2143 |  |  |  |  |
| QUIN | 0.1841 | 0.504 | 0.5147 | -0.1576 | 0.2294 | 0.1322 | 0.7165 | -0.1045 | 0.6307 | 0.5946 |  |  |  |  |
| KYNA | -0.2209 | 0.0957 | -0.2647 | 0.0482 | 0 | 0.8795 | 0.8061 | 0.05971 | 0.3571 | 0.8571 |  |  |  |  |
| QUIN/KYNA | 0.4148 | 0.2283 | 0.6667 | -0.3133 | 0.4472 | -0.3133 | -0.4414 | -0.1471 | 0.25 | -0.3929 |  |  |  |  |

**Supplementary Table 5.** Correlations within the pMCAo group between TRP (Tryptophan), KYN (Kynurenine), IDO (indoleamine 2,3 dioxygenase) activity, QUIN (Quinolinic acid), KYNA (Kynurenic acid), QUIN/KYNA serum levels and distance mouse travelled in open field, marble burying test, splash test, retention and reversal phases of the Barnes maze (*P <* 0.05 in green; not significant uncoloured). BM, Barnes maze.

| ***p* value** | | **Open Field distance** | **Marble**  **burying**  **test** | | **Splash test** | | **BM retention** | | | **BM reversal phase D11** | | |  |  |  |
| --- | --- | --- | --- | --- | --- | --- | --- | --- | --- | --- | --- | --- | --- | --- | --- |
| TRP | | 0.3268 | 0.8301 | | 0.2431 | | 0.4279 | | | 0.9349 | | |  |  |  |
| KYN | | 0.9768 | 0.8872 | | 0.1966 | | 0.9349 | | | 0.8401 | | |  |  |  |
| IDO | | 0.2431 | 0.3786 | | 0.9768 | | 0.6191 | | | 0.6191 | | |  |  |  |
| QUIN | | 0.6646 | 0.8401 | | 0.3266 | | 0.7930 | | | 0.9768 | | |  |  |  |
| KYNA | | 0.0476 | 0.7369 | | 0.6735 | | 0.6941 | | | 0.7172 | | |  |  |  |
| QUIN/KYNA | | 0.7930 | 0.2548 | | 0.1511 | | 0.1966 | | | 0.9349 | | |  |  |  |
|  | |  |  | |  | |  | |  | | |  | | |  |
|  | |  |  | |  | |  | |  | | |  | | |  |
| **r coefficient** | | **Open**  **Field distance** | **Marble**  **burying**  **test** | | **Splash test** | | **BM retention** | | | **BM reversal phase D11** | | |  |  |  |
| TRP | | 0.4048 | ­-0.09759 | | 0.4762 | | -0.3333 | | | 0.04762 | | |  |  |  |
| KYN | | 0.02381 | 0.07319 | | 0.5238 | | 0.4767 | | | 0.09524 | | |  |  |  |
| IDO | | -0.4762 | 0.366 | | 0.02381 | | 0.2143 | | | -0.2143 | | |  |  |  |
| QUIN | | -0.1905 | 0.1464 | | 0.4048 | | 0.3984 | | | 0.2462 | | |  |  |  |
| KYNA | | -0.7306 | 0.1473 | | -0.1796 | | 0.1676 | | | -0.1557 | | |  |  |  |
| QUIN/KYNA | | 0.119 | 0.4636 | | 0.5714 | | -0.5258 | | | 0.04762 | | |  |  |  |
|  | |  |  | |  | |  | |  | | |  | | |  |
|  |  | |  |  | |  | |  | | |  | | |  | |

**Supplementary Table 6.** Correlations within the pMCAo group between TRP (Tryptophan), KYN (Kynurenine), IDO (indoleamine 2,3 dioxygenase) activity, QUIN (Quinolinic acid), KYNA (Kynurenic acid), QUIN/KYNA serum levels and neuronal death, microglia/macrophages infiltration, and white matter density. Ipsi, ipsilateral; Hip, hippocampus; Contra, contralateral; MBP, Myelin Basic Protein (*P <* 0.05 in green; not significant uncolored).

| ***p* value** | **Fluorojade ipsi Hip.** | **Fluorojade contra Hip.** | **Iba1**  **peri infarct** | **Iba1**  **contra infarct** | **Iba-1 ipsi striatum** | **Iba1**  **contra striatum** | **Iba1**  **ipsi Hip.** | **Iba-1**  **contra Hip.** | **MBP**  **ipsi Striatum** | **MBP**  **contra Striatum** |  |  |  |  |
| --- | --- | --- | --- | --- | --- | --- | --- | --- | --- | --- | --- | --- | --- | --- |
| TRP | 0.882 | 0.882 | 0.9999 | 0.1248 | 0.9999 | 0.0589 | 0.9063 | 0.4867 | 0.75 | 0.9167 |  |  |  |  |
| KYN | 0.7033 | 0.7033 | 0.4976 | 0.0595 | 0.9194 | 0.5517 | 0.4444 | 0.0757 | 0.9167 | 0.75 |  |  |  |  |
| IDO | 0.752 | 0.882 | 0.6615 | 0.6932 | 0.3556 | 0.0857 | 0.1342 | 0.1095 | 0.75 | 0.9999 |  |  |  |  |
| QUIN | 0.3599 | 0.2675 | 0.7131 | 0.4976 | 0.4972 | 0.4618 | 01095. | 0.0519 | 0.3333 | 0.9167 |  |  |  |  |
| KYNA | 0.1625 | 0.0521 | 0.9857 | 0.6418 | 0.6583 | 0.7997 | 0.9999 | 0.8457 | 0.3333 | 0.75 |  |  |  |  |
| QUIN/KYNA | 0.882 | 0.882 | 0.6615 | 0.8063 | 0.5639 | 0.5206 | 0.048 | 0.0131 | 0.9999 | 0.75 |  |  |  |  |
|  |  |  |  |  |  |  |  |  |  |  |  |  |  |  |
|  |  |  |  |  |  |  |  |  |  |  |  |  |  |  |
| **r coefficient** | **Fluorojade ipsi Hip.** | **Fluorojade contra Hip.** | **Iba1**  **peri infarct** | **Iba1**  **contra infarct** | **Iba1 ipsi striatum** | **Iba-1**  **contra striatum** | **Iba1**  **ipsi Hip.** | **Iba-1**  **contra Hip.** | **MBP**  **ipsi striatum** | **MBP**  **contra striatum** |  |  |  |  |
| TRP | 0.07143 | 0.07143 | 0 | 0.5988 | -0.02857 | 0.6667 | -0.07143 | 0.2874 | -0.4 | -0.2 |  |  |  |  |
| KYN | 0.1667 | 0.1667 | -0.3214 | 0.7066 | 0.08571 | 0.2333 | 0.3571 | 0.6707 | -0.2 | 0.4 |  |  |  |  |
| IDO | 0.1429 | 0.07143 | -0.2143 | 0.1677 | 0.4857 | -0.6167 | 0.5868 | 0.6786 | 0.4 | 0 |  |  |  |  |
| QUIN | -0.381 | -0.4524 | 0.1786 | 0.3214 | 0.3714 | 0.3095 | 0.6786 | 0.7186 | 0.8 | -0.2 |  |  |  |  |
| KYNA | -0.5509 | -0.7186 | -0.01802 | 0.1928 | 0.2571 | -0.1004 | 0 | 0.0834 | 0.8 | -0.4 |  |  |  |  |
| QUIN/KYNA | -0.07143 | -0.07143 | 0.2143 | -0.1078 | 0.3143 | 0.25 | 0.7857 | 0.8383 | 0 | 0.4 |  |  |  |  |

**Supplementary Table 7.** Correlations within the pMCAo+D group between TRP (Tryptophan), KYN (Kynurenine), IDO (indoleamine 2,3 dioxygenase) activity, QUIN (Quinolinic acid), KYNA (Kynurenic acid), QUIN/KYNA serum levels and distance mouse travelled in open field, marble burying test, splash test, retention and reversal phases of the Barnes maze (*P <* 0.05 in green; not significant uncoloured). BM, Barnes maze.

| ***p* value** | | **Open Field distance** | **Marble**  **burying**  **test** | | **Splash test** | | **BM retention** | | | **BM reversal phase D11** | | |  |  |  |
| --- | --- | --- | --- | --- | --- | --- | --- | --- | --- | --- | --- | --- | --- | --- | --- |
| TRP | | 0.8820 | 0.8264 | | 0.3536 | | 0.3904 | | | 0.8401 | | |  |  |  |
| KYN | | 0.4279 | 0.7665 | | 0.3024 | | 0.2835 | | | 0.4618 | | |  |  |  |
| IDO | | 0.6646 | 0.7972 | | 0.5948 | | 0.4015 | | | 0.2162 | | |  |  |  |
| QUIN | | 0.2431 | 0.0450 | | 0.9635 | | 0.7838 | | | 0.1710 | | |  |  |  |
| KYNA | | 0.2431 | 0.1228 | | 0.8397 | | 0.8537 | | | 0.2992 | | |  |  |  |
| QUIN/KYNA | | 0.5364 | 0.7565 | | 0.6976 | | 0.8865 | | | 0.8401 | | |  |  |  |
|  | |  |  | |  | |  | |  | | |  | | |  |
|  | |  |  | |  | |  | |  | | |  | | |  |
| **r coefficient** | | **Open**  **Field distance** | **Marble**  **burying**  **test** | | **Splash test** | | **BM retention** | | | **BM reversal phase D11** | | |  |  |  |
| TRP | | 0.7143 | 0.69639 | | 0.4286 | | 0.3264 | | | -0.69524 | | |  |  |  |
| KYN | | 0.3333 | 0.1325 | | 0.4643 | | 0.4017 | | | 0.3095 | | |  |  |  |
| IDO | | 0.1905 | 0.1084 | | -0.25 | | 0.318 | | | 0.5 | | |  |  |  |
| QUIN | | 0.4762 | 0.735 | | 0.3571 | | 0.1088 | | | 0.5476 | | |  |  |  |
| KYNA | | 0.4762 | 0.6025 | | 0.1071 | | 0.67531 | | | -0.08827 | | |  |  |  |
| QUIN/KYNA | | -0.2619 | -0.1325 | | -0.3214 | | 0.05858 | | | -0.09624 | | |  |  |  |
|  | |  |  | |  | |  | |  | | |  | | |  |
|  |  | |  |  | |  | |  | | |  | | |  | |

**Supplementary Table 8.** Correlations within the pMCAo+D group between TRP (Tryptophan), KYN (Kynurenine), IDO (indoleamine 2,3 dioxygenase) activity, QUIN (Quinolinic acid), KYNA (Kynurenic acid), QUIN/KYNA serum levels and neuronal death, microglia/macrophages infiltration, and white matter density. Ipsi, ipsilateral; Hip, hippocampus; Contra, contralateral; MBP, Myelin Basic Protein (*P <* 0.05 in green; not significant uncolored).

| ***p* value** | **Fluorojade ipsi Hip.** | **Fluorojade contra Hip.** | **Iba1**  **peri infarct** | **Iba1**  **contra infarct** | **Iba-1 ipsi striatum** | **Iba1**  **contra striatum** | **Iba1**  **ipsi Hip.** | **Iba-1**  **contra Hip.** | **MBP**  **ipsi Striatum** | **MBP**  **contra Striatum** |  |  |  |  |
| --- | --- | --- | --- | --- | --- | --- | --- | --- | --- | --- | --- | --- | --- | --- |
| TRP | 0.1710 | 0.6191 | 0.793 | 0.4976 | 0.9999 | 0.9999 | 0.6500 | 0.6937 | 0.4444 | 0 |  |  |  |  |
| KYN | 0.9349 | 0.0831 | 0.2431 | 0.9999 | 0.9194 | 0.3444 | 0.7333 | 0.5302 | 0.3024 | 0.9999 |  |  |  |  |
| IDO | 0.0218 | 0.015 | 0.1966 | 0.6615 | 0.4972 | 0.5572 | 0.3889 | 0.2992 | 0.8397 | 0.7435 |  |  |  |  |
| QUIN | 0.5821 | 0.6191 | <0.9999 | 0.9635 | 0.8028 | 0.08827 | 0.3278 | 0.9206 | 0.9063 | 0.6134 |  |  |  |  |
| KYNA | 0;3599 | 0.2431 | 0.7520 | 0.7131 | 0.2417 | 0.9222 | 0.6500 | 0.0413 | 0.0341 | 0.8801 |  |  |  |  |
| QUIN/KYNA | 0.1323 | 0.1511 | 0.8401 | 0.6560 | 0.3558 | 0.9222 | 0.2449 | 0.0286 | 0.1389 | 0.8100 |  |  |  |  |
|  |  |  |  |  |  |  |  |  |  |  |  |  |  |  |
|  |  |  |  |  |  |  |  |  |  |  |  |  |  |  |
| **r coefficient** | **Fluorojade ipsi Hip.** | **Fluorojade contra Hip.** | **Iba1**  **peri infarct** | **Iba1**  **contra infarct** | **Iba1 ipsi striatum** | **Iba-1**  **contra striatum** | **Iba1**  **ipsi Hip.** | **Iba-1**  **contra Hip.** | **MBP**  **ipsi striatum** | **MBP**  **contra striatum** |  |  |  |  |
| TRP | -0.5476 | 0.2143 | 0.119 | -0.3214 | -0.02857 | 0 | 0.2319 | 0.1818 | -0.3571 | 0 |  |  |  |  |
| KYN | -0.04762 | 0.6667 | 0.4762 | 0 | 0.08571 | 0.4768 | 0.1739 | 0.291 | -0.4643 | 0 |  |  |  |  |
| IDO | 0.8095 | 0.8333 | 0.5238 | 0.2143 | 0.3714 | 0.3237 | -0.4348 | 0.5092 | 0.1072 | 0.1333 |  |  |  |  |
| QUIN | 0.2381 | 0.2143 | 0 | -0.03571 | -0.1429 | 0.9222 | -0.4928 | -0.65455 | -0.07143 | 0.2 |  |  |  |  |
| KYNA | -0.381 | -0.4762 | -0.1429 | 0.1786 | -0.6 | -0.08827 | 0.2319 | -0.8061 | 0.8234 | 0.6667 |  |  |  |  |
| QUIN/KYNA | 0.5952 | 0.5714 | 0.0924 | -0.2857 | 0.4857 | -0.08127 | -0.5798 | -0.5798 | -0.6429 | -0,1 |  |  |  |  |
